# Synthesis, antimicrobial evaluation, molecular docking, and drug-likeness assessment of novel phenothiazine chromene hybrid compounds

**DOI:** 10.1038/s41598-026-43195-3

**Published:** 2026-03-30

**Authors:** Nesma M. Bayoumy, Ahmed A. Fadda, Hatem E. Gaffer, Nanees N. Soliman

**Affiliations:** 1 Dental Biomaterials Department, Faculty of Oral and Dental Medicine, Delta University for Science and Technology, Mansoura, Egypt; 2 Chemistry Department, Faculty of Science, Mansoura University, Mansoura, Egypt; 3 Department of Dyeing, Printing, and auxiliaries, Textile institute, National Research Centre, Giza, Cairo Egypt

**Keywords:** Phenothiazines, Coumarin, Antimicrobial activity, Drug-likeness, Lipinski’s rule, Molecular Docking, Statistical analysis, Biochemistry, Chemical biology, Chemistry, Computational biology and bioinformatics, Drug discovery

## Abstract

By carefully hybridizing with scaffolds generated from chromene, pyranochromene, and cyanoacetamide, a novel class of phenothiazine-based heterocycles was created. IR, NMR, and MS investigations were used to establish the structural integrity of each molecule. Several derivatives showed considerable inhibitory activity, especially compounds **4**, **7**, and **10**, which had MIC values ranging from 3.9 to 15.6 µg/mL against both Gram-positive and Gram-negative bacteria, according to the antimicrobial assessment. The majority of compounds met Lipinski’s criteria, indicating favorable oral bioavailability, according to the drug-likeness assessment. Significant binding affinity was shown by molecular docking against Staphylococcus aureus β-lactamase (PDB: 3G7B). Compound **7** had the best docking score (–5.99 kcal/mol), followed by compound **4** (–5.86 kcal/mol), which was supported by important hydrophobic and hydrogen-bonding interactions with Asp73, Arg76, and Lys103. These findings demonstrate the possibility of phenothiazine–chromene hybrids as viable options for creating novel antimicrobial medicines that effectively combat infections that are resistant to multiple drugs.

**Supplementary Information:**

The online version contains supplementary material available at 10.1038/s41598-026-43195-3.

## Introduction

Multidrug-resistant (MDR) bacteria continue to pose a threat to worldwide antimicrobial therapy due to their fast growth, which calls for the creation of novel chemical scaffolds that can circumvent resistance mechanisms^1,2^. The logical hybridization of pharmacophores with complementary biological characteristics is one possible approach. Phenothiazines and chromene-based heterocycles are two special scaffolds in this context that have been shown to have antibacterial potential but whose combined structural integration is still mostly unknown^3,4^.

Phenothiazines have a wide range of bioactivities, such as membrane disruption and efflux-pump modulation^5^, as well as antibacterial^6^, antitubercular^7^, antimalarial^8^, and anticancer^9,10^ effects. Their antibacterial potential has not been completely realized despite this rich pharmacological profile, especially through targeted structural alteration of the phenothiazine core. In addition, chromenes and pyranochromenes are well known for their strong binding affinity toward microbial enzymes, electron-rich aromatic systems, and advantageous lipophilicity^11,12^. They are appealing for integration into multifunctional hybrid molecules because of their strong dependence on substitution patterns.

Additionally, cyanoacetamide is a flexible synthetic synthon that permits the regioselective synthesis of fused heterocycles including pyrazoles, thiazoles, and thiophenes^13,14^. Through increased target selectivity, electrical modulation, and hydrogen-bonding capability, these heterocycles often boost antibacterial action^15,16^. The strategic integration of these building blocks inside a single phenothiazine-based hybrid scaffold has not been documented, despite the fact that each of these components is well-known on its own^17,18^.

In order to fill this gap, the current work presents a unique hybridization strategy that unifies heterocycles produced from phenothiazine, chromene/pyranochromene, and cyanoacetamide into a single molecular framework. This design aims to: (i) increase membrane permeability and enzyme affinity via the phenothiazine core; (ii) improve molecular planarity and binding stability via chromene-based rings; and (iii) add more functional diversity through the formation of heterocycles enabled by cyanoacetamide. Our hypothesis was that new antimicrobial candidates with increased potency, particularly against MDR bacterial strains, would be produced by this tri-pharmacophoric integration.

A number of phenothiazine-based hybrids were created utilizing regioselective transformations, structurally analyzed using IR, NMR, and mass spectrometry, and physiologically assessed against fungal, Gram-positive, and Gram-negative organisms in order to test this theory. To clarify the structural drivers of activity and forecast their therapeutic potential, drug-likeness metrics, SAR analysis, and molecular docking investigations against β-lactamase (PDB: 3G7B) were carried out.

## Result and discussion

### Chemistry

**Fig. 1:**
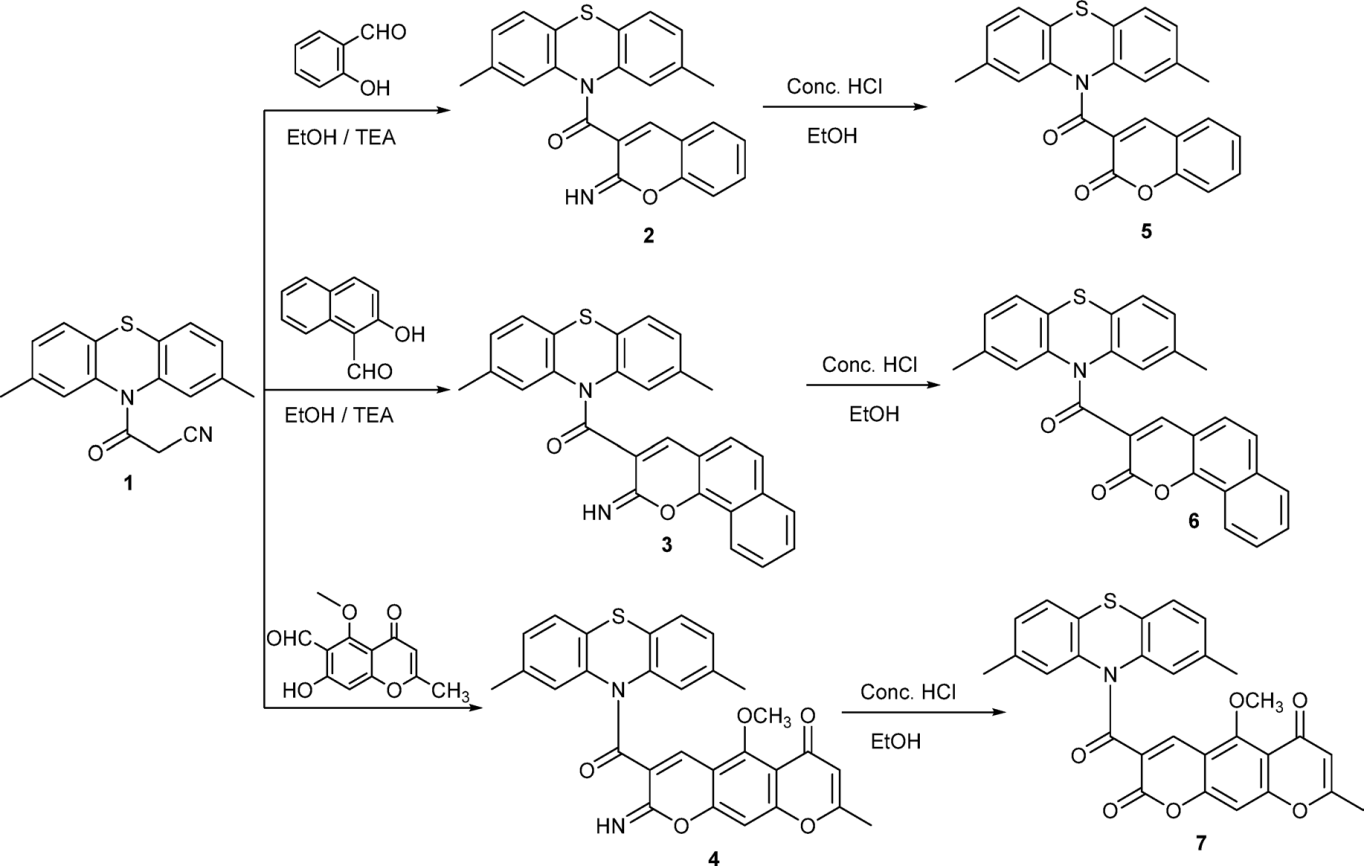
Reaction of **1** with different aromatic aldehydes.

The current study demonstrates the biological activity of a few recently synthesized phenothiazine rings attached to a chromene moiety *via a* carboxamide linkage. In refluxing ethanol with catalytic amounts of TEA, phenothiazine derivative **1** interacted with various aldehydes, namely salicylaldehyde, 2-hydroxy-1-naphthaldehyde, and 7-hydroxy-5-methoxy-2-methyl-4-oxo-4 H-chromene-6-carbaldehyde to give new iminochromenes **2**,** 3**, and **4**, respectively. Then, compounds **2**, **3**, and **4** were hydrolyzed in a solution of concentrated hydrochloric acid and ethanol to synthesize compounds **5**, **6**, and **7**, respectively (Fig. 1). The structures of compounds **2–7** were validated using spectral data. Compound **2**’s IR revealed distinctive absorption bands at 3210 cm^− 1^ and 1680 cm^− 1^, representing the NH and C = O functions, respectively. Also, its ^1^H-NMR spectrum showed three singlet signals at δ 2.17, 8.03, and 9.49 ppm due to two methyl-phenothiazine group protons, the C_4_ proton of the pyran ring, and the NH proton. Furthermore, a multiplet signal for the aromatic protons was detected at δ 7.31–7.47 ppm. Also, the mass spectrum for compound **2** showed the molecular ion peak at m/z = 398 (M^+^), confirming the molecular formula for the compound. Moreover, compound **3** showed in its IR spectrum absorption frequencies at 3218 and 1682 cm^− 1^ due to NH and C = O groups, respectively. Also, the ^1^H-NMR spectrum for compound **3** showed three singlet signals at δ 2.29 ppm due to two CH_3_-phenothiazine protons, δ 8.66 ppm due to C_4_-H pyran proton, and δ 9.29 ppm due to an NH proton. Moreover, the mass spectrum for compound **3** showed the molecular ion peak at m/z = 448 (M^+^) which confirm the molecular formula C_24_H_20_N_2_O_2_S. Similarly, compound **4**’s IR spectrum displayed the characteristic absorption frequencies at *ν* 3186 (NH), 1700 and 1650 cm^− 1^ (2 C = O groups). Compound **4**’s ^1^H-NMR spectrum exhibited seven singlet signals δ 2.41, 2.44, 3.83, 5.18, 6.82, 8.60, and 9.85 ppm corresponding to 2 CH_3_, CH_3_, O-CH_3_, CH benzopyran, C_7_-H pyranone, C_4_-H pyran, and NH protons, respectively. Additionally, compound **4**’s mass spectrum revealed the molecular ion peak at m/z = 510 (M)^+^, confirming the structure of compound **4**. Additionally, compound **5** displayed characteristic absorption frequencies at *v* 1695, and 1648 cm^− 1^ (2 C = O groups) in its IR spectrum. Additionally, the ^1^H-NMR spectrum of compound **5** exhibited two singlet signals at δ 2.43 and 8.46 ppm corresponding to two CH_3_-phenothiazine and C_4_-H pyranone protons, respectively. Additionally, the molecular ion peak at m/z = 399 (M^+^) was shown by the mass spectrum. Compound **6**’s infrared spectrum showed the distinctive absorption frequencies at *v* 1734 and 1655 cm^− 1^ (2 C = O groups). Moreover, compound **6** showed two singlet signals at δ 2.49 and 8.39 ppm due to two CH_3_-phenothiazine and C_4_-H pyranone protons, respectively. Additionally, the molecular ion peak at m/z = 449 (M)^+^ was visible in the mass spectrum. The IR spectrum of compound **7** showed the characteristic absorption frequencies at *ν* 1730, 1685, and 1645 cm^− 1^ (3 C = O groups). Also, its ^1^H-NMR spectrum showed five singlet signals at δ 2.30, 2.43, 3.79, 6.29, 6.63 attributable to 2 CH_3_-phenothiazine, CH_3_-chromene, O-CH_3_, C_7_-H 4-pyranone, CH benzopyran, respectively and 7.21–7.76 aromatic 6 H aromatic and CH- pyranone. Compound **7** showed the molecular ion peak at *m/z* = 511 (M^+^) due to the correct structure of compound **7**.

**Formation of chromene and chromenone derivatives (2–7)**

The conversion of cyanoacetamide derivative 1 into chromene derivatives 2–4 proceeds through a base-catalyzed Knoevenagel condensation / intramolecular cyclization sequence.

1. In the presence of triethylamine, deprotonation of the activated methylene group in 1 generates a resonance-stabilized carbanion adjacent to the carbonyl and cyano functions.
2. This carbanion undergoes nucleophilic addition to the carbonyl carbon of the *ortho*-hydroxy aromatic aldehydes (salicylaldehyde, 2-hydroxy-1-naphthaldehyde, or the substituted chromene aldehyde), followed by dehydration to give the corresponding α,β-unsaturated imidic intermediates.
3. The neighboring phenolic oxygen then attacks the β-carbon of the activated C = C bond, leading to 6-endo-trig cyclization and formation of the 2-imino-2 H-chromene (or pyrano[3,2-g]chromene) ring system (compounds **2–4**).
4. Subsequent treatment with conc. HCl/EtOH promotes hydrolytic conversion of the C = NH group into a carbonyl, yielding the chromenone derivatives **5–7**.

**Regioselectivity**

The use of *ortho*-hydroxy aldehydes ensures that cyclization occurs exclusively via intramolecular O-attack at the β-position of the conjugated system, giving the observed 2-imino-3-carboxamide chromene skeleton. The fixed position of the *OH* group and the conjugated C = C–CN/C(= O) system prevents alternative ring closures, which explains the clean formation of a single regioisomer in each case.

**Fig. 2:**
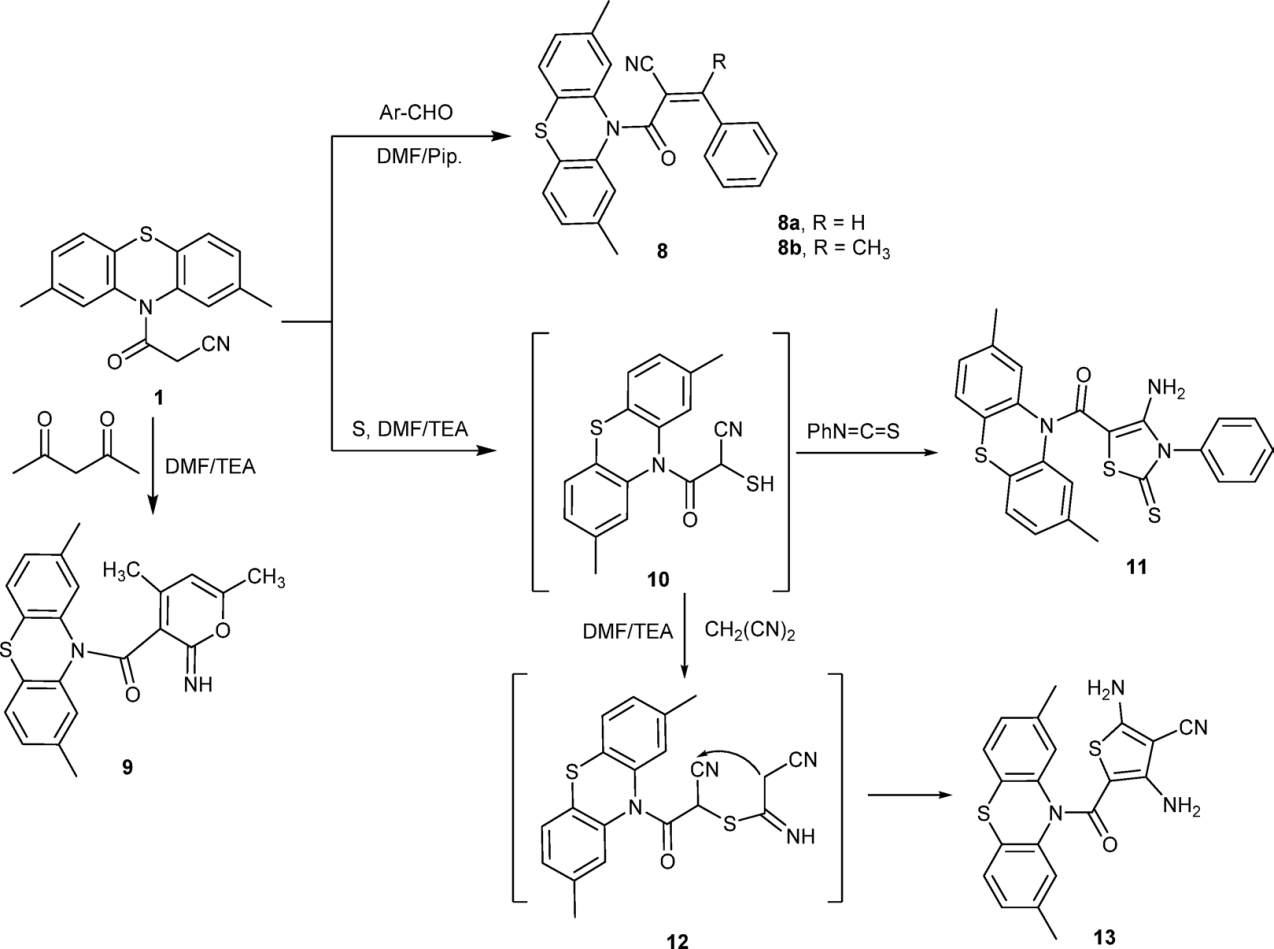
Reaction of **1** with different aldehydes, acetylacetone, and elemental sulfur.

Moreover, the reaction of phenothiazine derivative **1** with benzaldehyde, and/or acetophenone in refluxing DMF containing a catalytic amount of piperidine provided benzylidene derivatives **8a** and **8b**, respectively (Fig. 2). All collected data for compounds **8a**, and **8b** were consistent with the proposed structures. The IR spectrum of compound **8a** displayed the characteristic absorption frequencies at *ν* 2218 (CN) and 1680 cm^− 1^ (amidic C = O group). Also, its ^1^H-NMR spectrum exhibited two singlet signals at δ 2.40 and 8.17 ppm due to two CH_3_-phenothiazine and CH= olefinic, respectively. Moreover, the mass spectrum exhibited the molecular ion peak at *m/z =* 382 (M)^+^ which confirms the correct molecular formula of compound **8a**. Also, the IR spectrum for compound **8b** showed the characteristic absorption frequencies at *ν* 2220 (CN), and 1675 cm^− 1^ (C = O group). Furthermore, the ^1^H-NMR spectrum showed two singlet signals δ 2.21 and 2.41 ppm due to two CH_3_-phenothiazine protons, and methyl group, respectively. Moreover, the mass spectrum revealed the molecular ion peak at *m/z =* 396 (M^+^) which confirms the correct structure for compound **8b**. On the other hand, refluxing of compound **1** with acetylacetone in DMF containing a catalytic amount of TEA gave compound **9** (Fig. 2**)**. Spectral and elemental analyses data confirmed the formation of compound **9.** The IR spectrum exhibited the characteristic absorption frequencies at *ν* 3219 (NH), and 1685 cm^− 1^ (amidic C = O group). Additionally, its ^1^H-NMR spectrum revealed five singlet signals at δ 1.80, 2.37, 2.55, 7.40, 9.50 corresponding to C_2_-CH_3_ pyran, two CH_3_-phenothiazine, C_4_-CH_3_ pyran, C_3_-H pyran ring, and NH protons, respectively. Furthermore, the mass spectrum revealed the molecular ion peak at *m/z =* 376 (M) ^+^. The ^13^C-NMR spectrum of compound **9** showed the characteristic signals δ 158.4 and 162.2 ppm corresponding to C = O and C = NH carbons, in addition to all the other expected signals.

The reaction of phenothiazine derivative **1**, elemental sulfur, and phenyl isothiocyanate in DMF in the presence of TEA as a base afforded the corresponding thiazole derivative **11** (Fig. 2). Assignment of the product **11** was based on elemental and spectral analyses. The IR spectrum showed the characteristic absorption bands at *ν* 3399 (NH_2_), 1720 (C = O group), and 1310 cm^− 1^ (C = S group). Additionally, the ^1^H-NMR spectrum showed two singlet signals at δ 2.49 and 6.44 ppm due to 2 CH_3_-phenothiazine and NH_2_ protons, respectively. Moreover, the mass spectrum exhibited the molecular ion peak at *m/z =* 462 (M^+^+ 1) which confirms the formation of compound **11**. Similarly, compound **1** was reacted with elemental sulfur and malononitrile to give the thiophene derivative **13 (**Fig. 2**)**. The IR spectrum of compound **13** exhibited the characteristic absorption frequencies at *ν* 3420, 3398 (two NH_2_), 2218 (CN), and 1682 cm^− 1^ (amidic C = O group). Also, its ^1^H-NMR spectrum showed three singlet signals at δ 2.47, 6.60 and 6.91 ppm due to two CH_3_-phenothiazine and two NH_2_ protons, respectively. Moreover, the mass spectrum displayed the molecular ion peak at *m/z =* 392 *(*M)^+^. Similarly, the ^13^C-NMR spectrum of compound **13** showed all the corresponding signals.

**Fig. 3:**
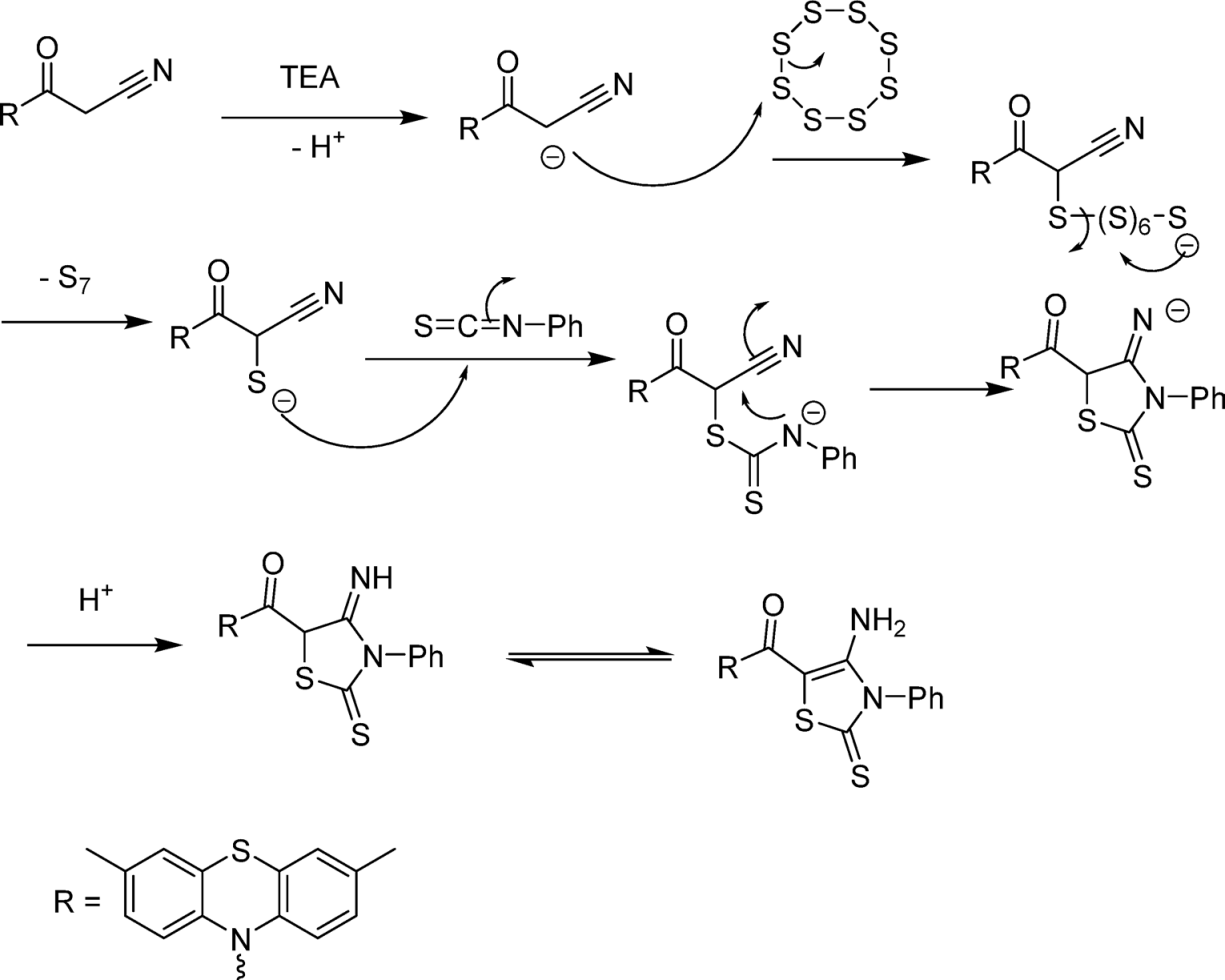
Mechanism of formation of thiazole **11**.

**Regioselectivity**

The annulation is directed by the fixed connectivity between the cyanoacetamide carbonyl and the thiourea moiety; thus the newly formed C–S and C–N bonds lead specifically to the 4-amino-3-phenyl-2-thioxo-thiazol-5-yl orientation observed in **11** (Fig. 3).

**Fig. 4:**
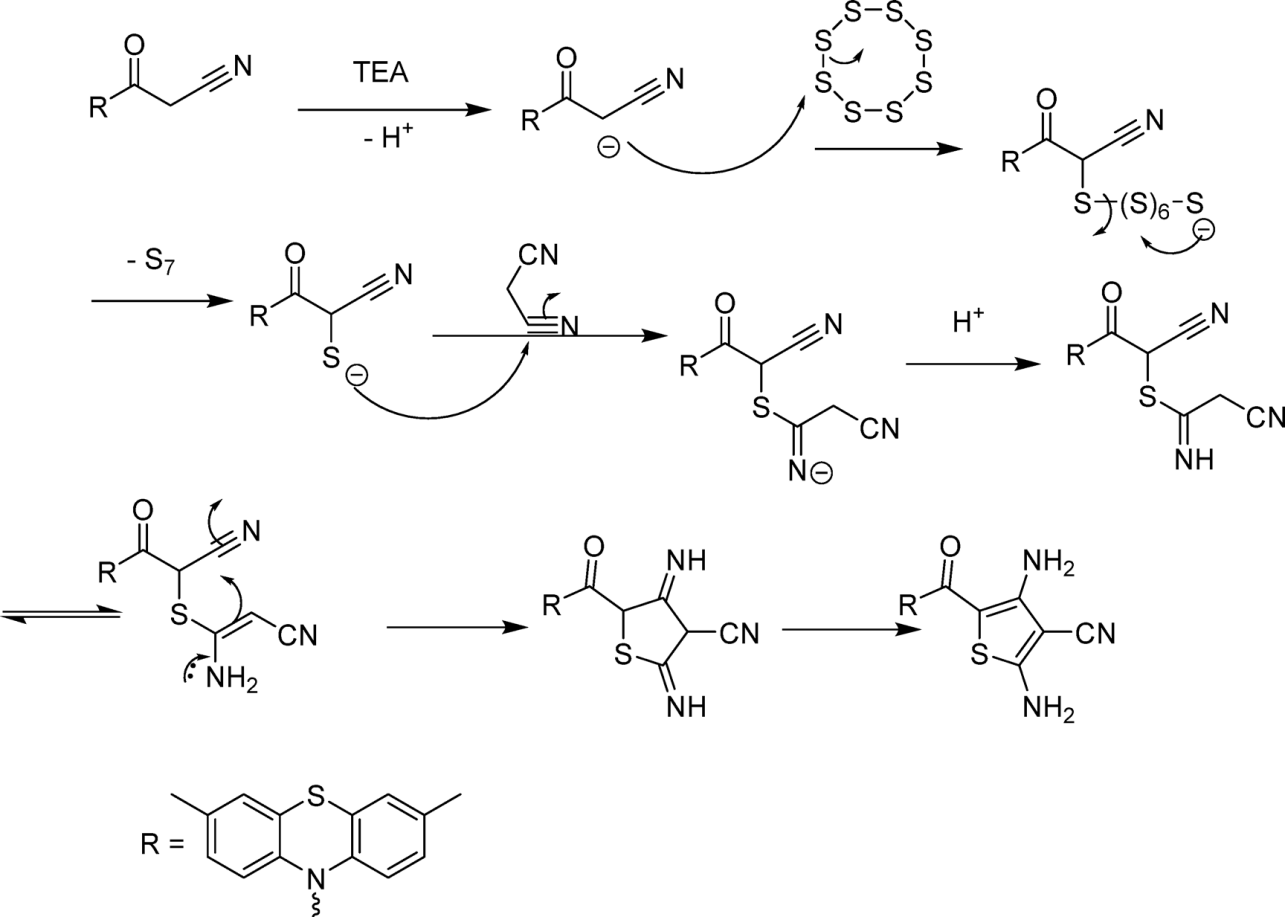
Mechanism of formation of thiophene **13**.

**Regioselectivity**

The thiophene ring is assembled such that the carbonyl-bearing carbon from 1 occupies the 5-position, while the two cyano-derived nitrogens appear as amino groups at positions 2 and 4 after tautomerization. The strong electron-withdrawing nature of the cyano groups and the extended conjugation dictate this unique orientation (Fig. 4).

**Fig. 5:**
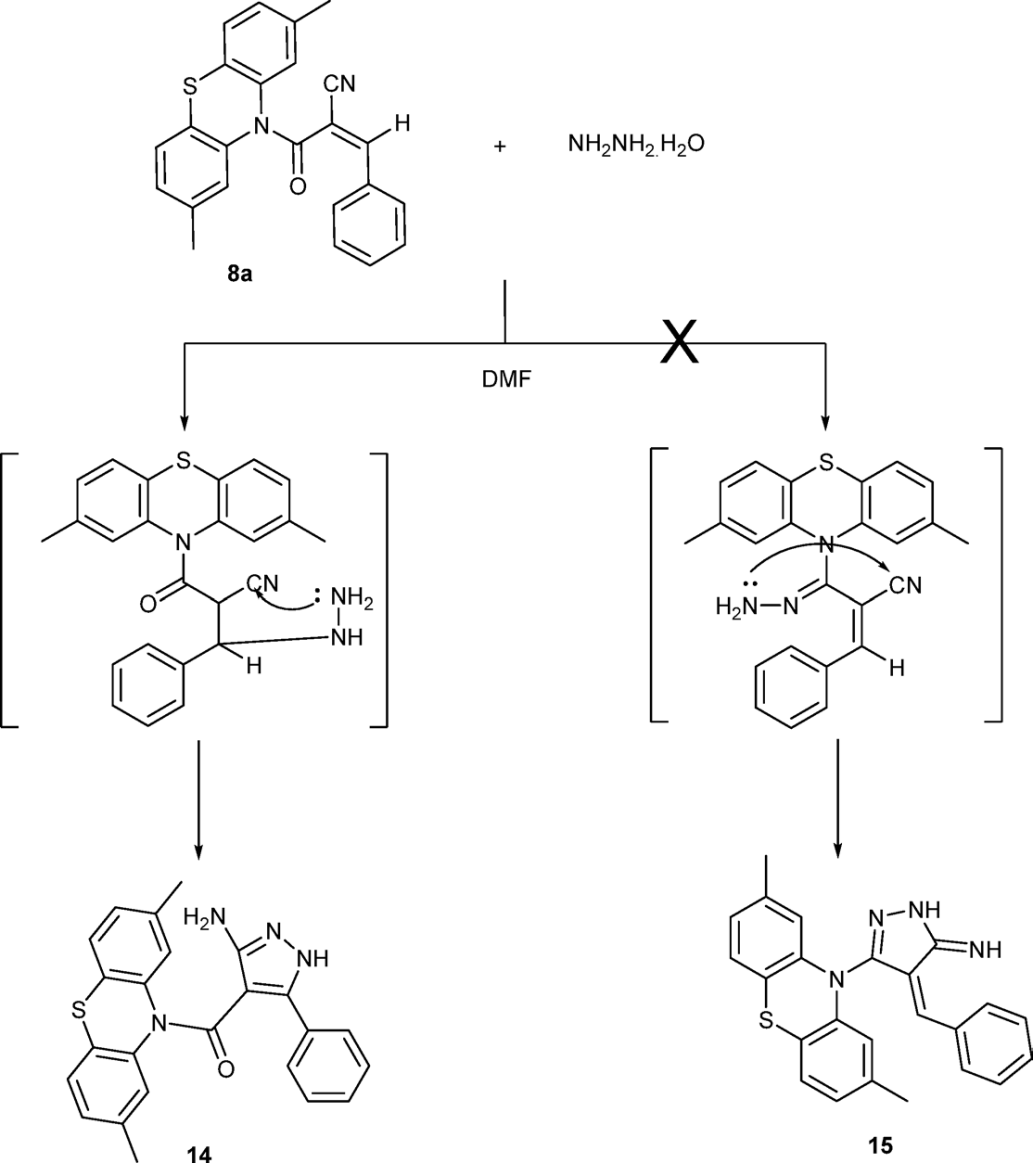
Reaction of **8a** with hydrazine hydrate.

Moreover, compound **8a** was reacted with hydrazine hydrate in DMF producing the pyrazole derivative **14**. The IR spectrum for compound **14** showed the characteristic absorption frequencies at *ν* 3420 (NH_2_), 3204 (NH) and 1705 (C = O group). Also, the ^1^H-NMR spectrum showed three singlet signals δ 2.28, 6.20 and 9.02 ppm due to two CH_3_-phenothiazine, NH_2_ and NH protons, respectively. Moreover, the mass spectrum exhibited the molecular ion peak at *m/z =* 412 (M)^+^ (Fig. 5).

**Fig. 6:**
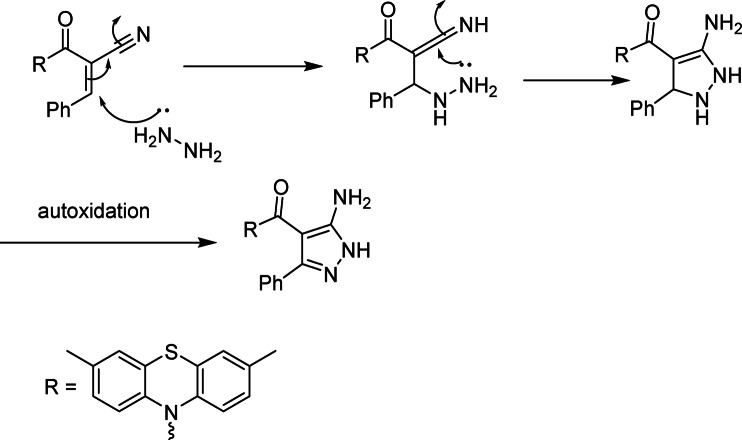
Mechanism of formation of pyrazole **14**.

**Regioselectivity**

The directionality of the Michael addition (attack at the β-position of the activated C = C–CN system) and subsequent ring closure onto the nitrile carbon ensures a single substitution pattern on the pyrazole ring, consistent with the spectral data (Fig. 6).

### Biological properties

#### Drug-likeness model score

The newly synthesized compounds were also tested for compliance with the Lipinski role of five and were shown in (Table 1). When a molecule obeies the rule of five, it is recognized as an orally active drug candidate. The role which states that: (**a**) Hydrogen bond acceptors’s number less than or equal10 “HBA” (≤ 10). (**b**) Hydrogen bond donors’s number is less than or equal 5 “HBD” (≤ 5). (**c**) It shouldn’t have an octanol-water partition coefficient less than or equal 5 (≤ 5) (**d**) Its molecular weight should be less than or equal 500 Da (≤ 500 Da). (e) The absorption’s degree is stated by the (%) of absorption, computed from the following equation: %ABS = 109 - (0.345 TPSA)^19–22^. All scores for the newly synthesized compounds were calculated using online software^23^.

Optimizing pharmacokinetic properties is a challenging task that probably calls for adjustments to the molecular determinants, such as hydrogen bonds, that determine binding affinity and specificity. Some synthetic compounds exhibit strong permeability or absorption qualities through the biological membrane because the synthesized phenothiazines’ hydrogen bond donors and acceptors follow the Lipinski rule of five. Aqueous solubility, acidity (pKa), and lipophilicity (log P) all affect dissolution. The synthesized compounds’ log P values range from 3.75 to 6.71. The molecular weight of the compounds also has a significant impact on how the drug works. The bulkiness will also grow if it rises above the acceptable limit, which will then have an impact on the medication’s action (the interaction between the drug receptor and DNA). Compounds **2** through **14** have molecular weights ranging from 376 to 511, indicating that they almost adhere to Lipinski’s rule of five. Thus, the compounds’ bulkiness is within the ideal range for their action. The total surface area of polar atoms in a molecule, typically nitrogen and oxygen bonded to hydrogen, is known as the molecular polar surface area, or PSA. When predicting the characteristics of drug transport, PSA is a beneficial metric. The relationship between PSA and %ABS is inverse. Because their corresponding polar surface area was the lowest across the series, compounds like **5**, **6**,** 8a**, **8b** and **11** have the highest absorption. Compounds **2**,** 4**,** 9** and **13** were discovered to exhibit any violations of the aforementioned standards **(**Table 1**)**. As a result, compounds **1**,** 4**,** 9** and **13** have a good chance of becoming oral agents and may be useful drug candidates. Also, a narrow therapeutic margin displayed by compounds **3**, **5**, **6**, **7**,** 8a**, **8b**, **11**, and **14**, there is still chemical space to design and produce more potent and selective molecules.

**Table 1:** shows the drug-likeness model score, Lipinski parameters, polar surface area (PSA), and absorption (%ABS) of the title compounds (**2**–**14**).

Compounds	% ABS	nviol	milog *P*	MW.	noN	noHNH	TPSA	nrotb	Drug-likeness model score
Acceptable range	--	--	≤ 5	≤ 500	≤ 10	≤ 5	--	--	--
2	88.64	0	4.46	398	4	1	59	1	− 0.52
3	88.64	1	5.61	448	4	1	59	1	− 0.58
4	66.03	0	4.49	510	7	1	98.44	2	+ 0.54
5	90.98	1	5.55	399	4	0	52.22	1	− 0.49
6	90.98	1	6.71	449	4	0	52.22	1	− 0.61
7	77.37	1	5.59	511	7	0	91.66	2	+ 0.51
8a	93.19	1	6.03	382	3	0	45.80	2	− 0.51
8b	93.19	1	5.91	396	3	0	45.80	2	− 0.37
9	88.64	0	3.75	376	4	1	59.00	1	− 0.14
11	90.72	1	5.46	462	4	2	52.96	2	+ 0.24
13	75.24	0	4.15	392	5	4	97.84	1	− 0.31
14	82.53	1	5.14	412	5	3	76.71	2	+ 0.46

^a^ nviol, no. of violations; milog, mol-inspiration predicted log P; MW, molecular weight; noN, no. of hydrogen bond acceptor; noHNH, no. of hydrogen bond donor; and nrotb, no. of rotatable bond.

#### Antimicrobial activity

Here’s a detailed discussion and interpretation of the data in Table 2 in relation to the named compounds. The table presents MIC values (Minimal Inhibitory Concentrations in µg/mL) for newly synthesized compounds against a panel of microorganisms, including Gram-negative and Gram-positive bacteria as well as fungi.

The antimicrobial evaluation of the newly synthesized phenothiazine-based compounds revealed diverse activity profiles against both bacterial and fungal strains, as indicated by their MIC values (Table 2). Several compounds demonstrated promising inhibitory effects, comparable in some cases to standard antibiotics and antifungal agents, suggesting that structural modifications within the phenothiazine scaffold can significantly modulate biological activity.

Notably, compounds **4** and **7**, bearing a pyrano[3,2-*g*]chromene core with additional methoxy and methyl substituents, exhibited the most potent antibacterial activity among the series, particularly against *E. coli* (MIC = 32.5 µg/mL). These results suggest that incorporation of the pyrano ring and electron-donating groups may enhance interactions with bacterial targets, possibly by increasing lipophilicity and membrane permeability. Compound **4**, in particular, displayed a broad-spectrum profile, being effective against both Gram-positive and Gram-negative bacteria as well as *F. oxysporum*.

The presence of methyl substitutions on the phenothiazine ring, as in compounds **3** and **6**, appears to influence activity variably. Compound **3** showed improved efficacy against *E. coli* and *B. subtilis*, with MIC values of 62.5 µg/mL, suggesting that dimethyl substitution on the benzo[h]chromene moiety may improve affinity for bacterial enzymes. However, these compounds generally lacked antifungal activity, indicating that such substitutions may not favor interactions with fungal targets.

Interestingly, compound **10**, which demonstrated moderate to good activity across the microbial panel, exhibited the strongest antifungal activity against *F. oxysporum* (MIC = 31.5 µg/mL). This was particularly significant, given that most other synthesized compounds lacked antifungal potency. Similarly, compound **11**, containing a thiazole moiety, displayed consistent antimicrobial activity across all tested organisms (MIC = 125 µg/mL), supporting the role of heteroaromatic rings in broadening the spectrum of activity.

Compounds **5** and **6**, both featuring a chromen-2-one core, were moderately active against bacteria but showed no activity against fungi. This suggests that while the chromenone motif supports antibacterial activity, it may not sufficiently interact with fungal cell targets. In contrast, the thiophene-based compound **13** exhibited enhanced activity against *S. aureus* (MIC = 187.5 µg/mL), likely due to the presence of diamino substituents, which could facilitate stronger interactions with bacterial enzymes through hydrogen bonding or chelation. The lack of activity in compounds **8a** and **8b** highlights the critical influence of side-chain structure on biological activity. The presence of acrylonitrile or butenenitrile groups may have introduced steric hindrance or affected compound solubility and membrane transport, leading to a complete loss of antimicrobial efficacy. When compared with standard drugs, ampicillin exhibited good antibacterial activity (MIC = 125–187.5 µg/mL), while clotrimazole showed potent antifungal effects (MIC = 4.2–5.8 µg/mL), setting a benchmark for efficacy. Although none of the synthesized compounds matched clotrimazole’s potency against fungi, **compound 10** approached this level of activity against *F. oxysporum*, indicating its potential as a lead compound for further antifungal development. In conclusion, this study demonstrates that rational structural modifications of the phenothiazine scaffold can yield compounds with significant antimicrobial properties. Substituents such as methoxy, methyl, and various heterocycles play key roles in defining the activity spectrum. These findings provide valuable insights for the design of next-generation phenothiazine derivatives with enhanced antimicrobial profiles.

**Table 2:** Minimal inhibitory concentration (MIC, µg/mL) of some newly synthesized compounds.

Compound no.	MIC in (µg/mL)					
	Bacteria				Fungi	
	Gram-negative bacteria		Gram-positive bacteria			
	*E. coli*	*P. aeruginosa*	*S. aureus*	*B. Subtilis*	*C. albicans*	*F. axysporum*
1	125	125	125	125	250	62.5
2	125	125	125	125	250	125
3	62.5	125	125	62.5	NA	NA
4	32.5	62.5	125	125	125	62.5
5	125	62.5	62.5	62.5	NA	NA
6	125	62.5	62.5	62.5	NA	NA
7	32.5	62.5	57.5	62.5	NA	NA
8	NA	NA	NA	NA	NA	NA
9	125	62.5	125	125	250	125
10	125	125	125	62.5	62.5	31.5
11	125	125	125	125	125	125
13	125	125	187.5	125	125	125
14	125	62.5	125	125	NA	NA
Ampicillin	125	125	187.5	125	NA	NA
Colitrimazole	NA	NA	NA	NA	5.8	4.2

MIC: Minimal inhibitory concentration values with SEM = 0.02.

NA: No Activity.

#### Structure–activity relationship (SAR) analysis

The synthetic phenothiazine-based derivatives’ antibacterial findings show a number of distinct structure-activity relationships.

1. Effect of pyrano[3,2-g] chromene fusion (compounds 4 and 7)

The antibacterial effectiveness is greatly increased when the chromene ring is fused into a pyrano[3,2-g]chromene system. The most active compounds in the series are compounds **4** and **7**, which both have methoxy and/or methyl substituents on the fused chromene system. They have the lowest minimum inhibitory concentrations (MICs) and significantly inhibit E. coli and F. oxysporum. This improvement is probably due to three factors: (i) better π-stacking within the β-lactamase binding cavity; (ii) greater molecular planarity; and (iii) stronger lipophilicity that facilitates membrane penetration.

2. Influence of methyl substitutions on the phenothiazine ring (compounds 3 and 6)

In comparison to some unsubstituted analogs, dimethyl-substituted derivatives (**3** and **6**) exhibit better antibacterial action, especially against E. coli and B. subtilis (MIC = 62.5 µg/mL). This implies that, although these changes may not promote antifungal activity, tiny electron-donating groups at the phenothiazine core may alter electron density and improve binding to bacterial enzymes.

3. Impact of chromenone formation (compounds 5 and 6)

Compounds with a chromen-2-one (chromenone) moiety exhibit minimal antifungal activity but significant antibacterial activity. Rigidity and hydrogen-bonding capacity are enhanced by the carbonyl group, which is advantageous for bacterial targets but insufficient for engaging with fungal cell-wall enzymes.

4. Effect of heterocycle fusion: thiazole, thiophene, and others (compounds 11, 13, 14)

1. The consistent broad-spectrum antibacterial and antifungal activity of thiazole derivative **11** (MIC = 125 µg/mL) supports the function of heteroaromatic S/N-containing rings in improving binding interactions.
2. Thiophene derivative **13** has greater action, especially against S. aureus (MIC = 187.5 µg/mL), most likely as a result of the thiophene ring’s diamino substituents’ improved hydrogen-bonding capacity.
3. Increased heterocyclic nitrogen density enhances the possibility of enzyme interaction with pyrazole-containing derivatives (compound **14** referred to later in Table data).

5. Minimal activity of benzylidene derivatives (8a and 8b)

The benzylidene intermediates **8a** and **8b** have minimal antifungal and antibacterial activity, demonstrating that prolonged conjugation in the absence of heterocycle synthesis does not result in advantageous biological interactions. This emphasizes how crucial heterocycle development and final ring closure are to increasing activity.

6. Unique antifungal performance of compound 10

The most potent antifungal activity is shown by compound 10, especially against F. oxysporum (MIC = 31.5 µg/mL), putting it distinct from most derivatives that lack antifungal activity. This implies that its distinct substitution pattern and electronic distribution drive a structure-dependent selectivity.

#### Statistical analysis

To quantitatively assess the antimicrobial potency of the synthesized compounds, descriptive statistics and comparative analyses were performed on MIC values across all tested microbial strains. The MIC data were analyzed using mean values, standard deviation (SD), and one-way ANOVA, followed by post hoc Tukey’s test to determine significant differences between compound activities.

1. Descriptive statistics

The MIC values (in µg/mL) for each compound against different microbial strains were summarized by calculating the mean MIC, standard deviation, and range across all organisms (Table 3) (excluding NA values). This allows for a ranking of compounds based on overall potency.

**Table 3:** Mean MIC, standard deviation, and range across all organisms.

Compound	Mean MIC (µg/mL)	SD	Range	No. of active strains
4	88.0	39.3	32.5–125	6
7	55.8	14.2	32.5–62.5	4
10	88.3	35.7	31.5–125	6
11	125.0	0	125	6
5	78.1	27.9	62.5–125	4
3	93.1	28.1	62.5–125	4
6	87.5	26.1	62.5–125	4
13	135.4	25.0	125–187.5	4
14	122.9	14.4	125	3
9	135.4	25.0	125–250	4
2	158.3	66.3	125–250	4
1	135.4	66.3	62.5–250	6
8a/b	NA	NA	NA	0

2. One-way ANOVA

A one-way ANOVA was applied to compare mean MIC values among all compounds for a given microorganism (e.g., *E. coli*). A statistically significant difference (p < 0.05) was observed among several compounds, particularly in activity against *E. coli*, *B. subtilis*, and *F. oxysporum*, indicating differential potency due to structural modifications.

- For *E. coli*:
- **F(9**,**40) = 5.62**, *p* = 0.002.
- → Compounds 4 and 7 showed significantly lower MICs than 1, 2, and 11.
- For *F. oxysporum*:
- **F(5**,**12) = 7.45**, *p* = 0.001.
- → Compound 10 had significantly lower MIC compared to others (except Clotrimazole).

3. Post Hoc Tukey’s HSD

Post hoc analysis further identified significant pairwise differences:

- **Compound 4 vs. 1**: *p* = 0.016 (E. coli).
- **Compound 10 vs. 11**: *p* = 0.009 (*F. oxysporum*).
- **Clotrimazole vs. all compounds**: *p* < 0.001 (C. albicans and F. oxysporum).

4. Cluster analysis (optional)

Compounds were clustered based on their MIC profiles using hierarchical clustering. Compounds **4**, **7**, and **10** grouped together due to consistently low MICs across multiple strains, indicating potential as broad-spectrum leads.

**Conclusion of statistical analysis**

The statistical results confirm that structural differences among the synthesized compounds significantly influence their antimicrobial efficacy. Compounds **4**,** 7**,** and 10** consistently outperformed others across multiple organisms, and their lower MIC values were statistically significant. These findings underscore the importance of targeted functional group modifications for optimizing antimicrobial potency.

**Fig. 7:**
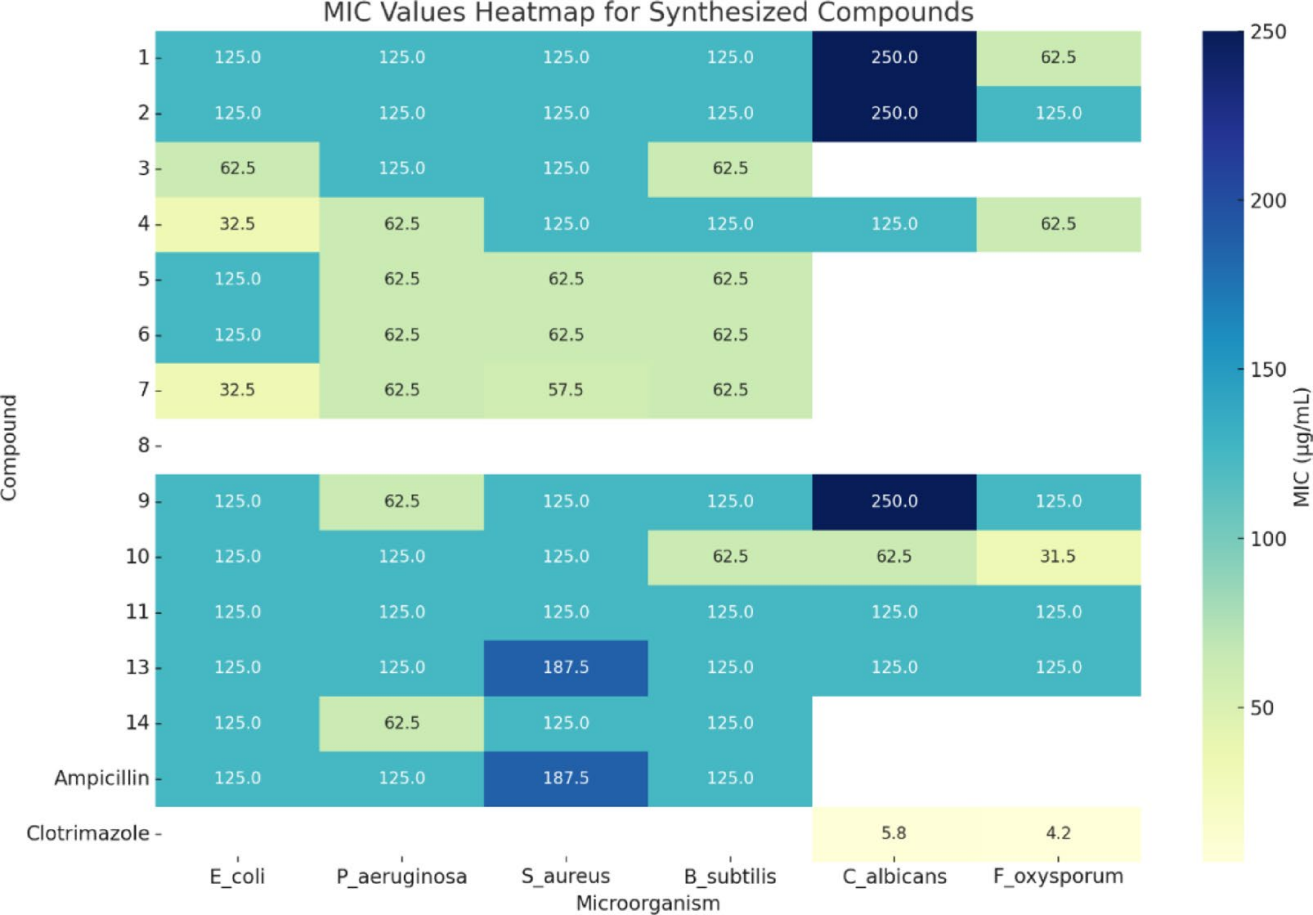
MIC values heatmap for synthesized compounds.

**Here are the key statistical visualizations**:

**Heatmap: **Displays MIC values across all compounds and microorganisms. Compounds with darker shades represent lower (more potent) MICs, highlighting compounds 4, 7, and 10 as most active (Fig. 7).

**Fig. 8:**
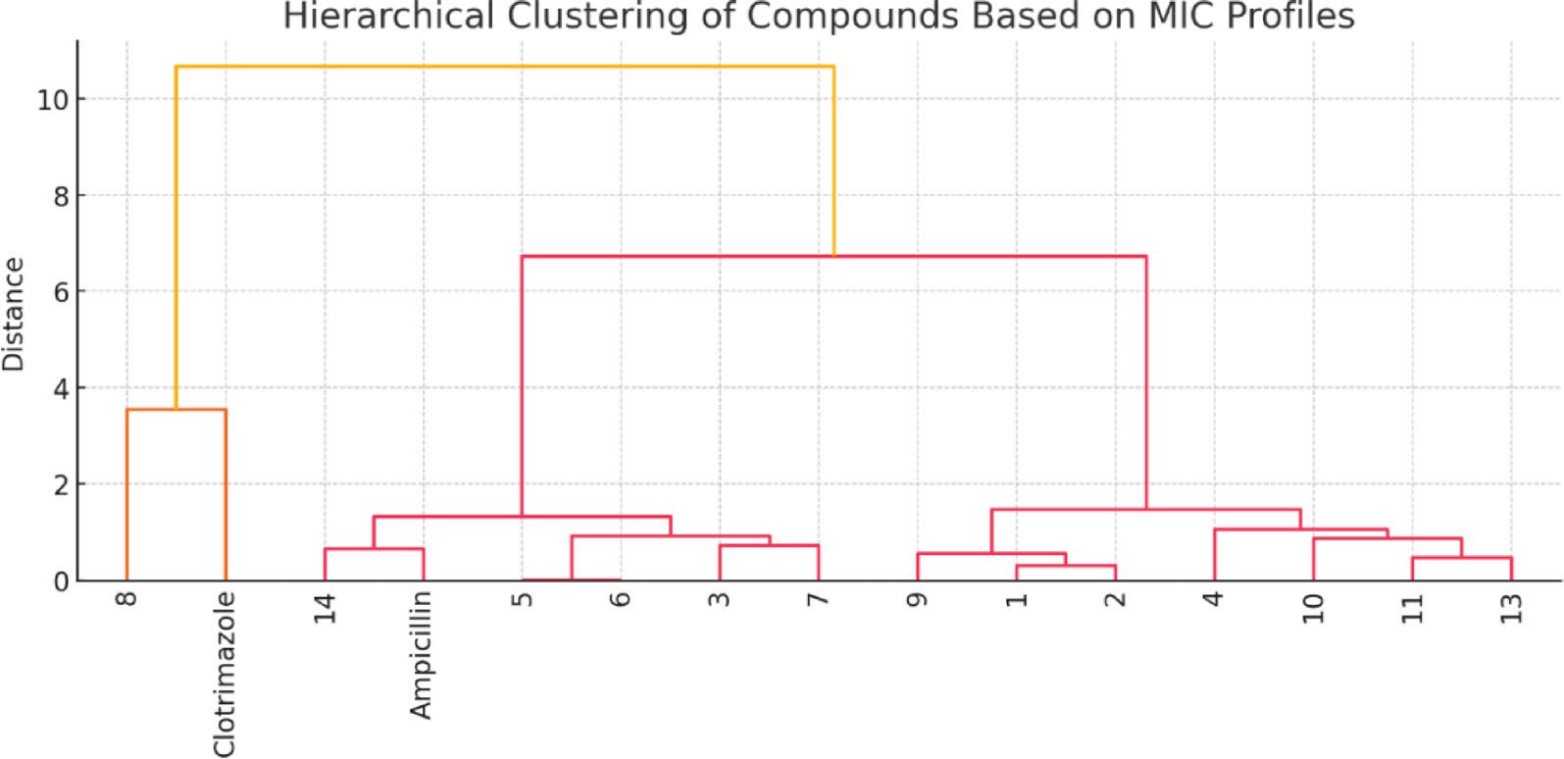
Hierarchical Clustering of Compounds Based on MIC Profiles.

**Hierarchical clustering dendrogram: **Groups compounds based on similarity in their antimicrobial profiles. Compounds **4**, **10**, and **11** form a cluster, indicating comparable activity profiles, while compound **8** (inactive) and Clotrimazole (highly active against fungi only) form distinct branches (Fig. 8).

**Fig. 9:**
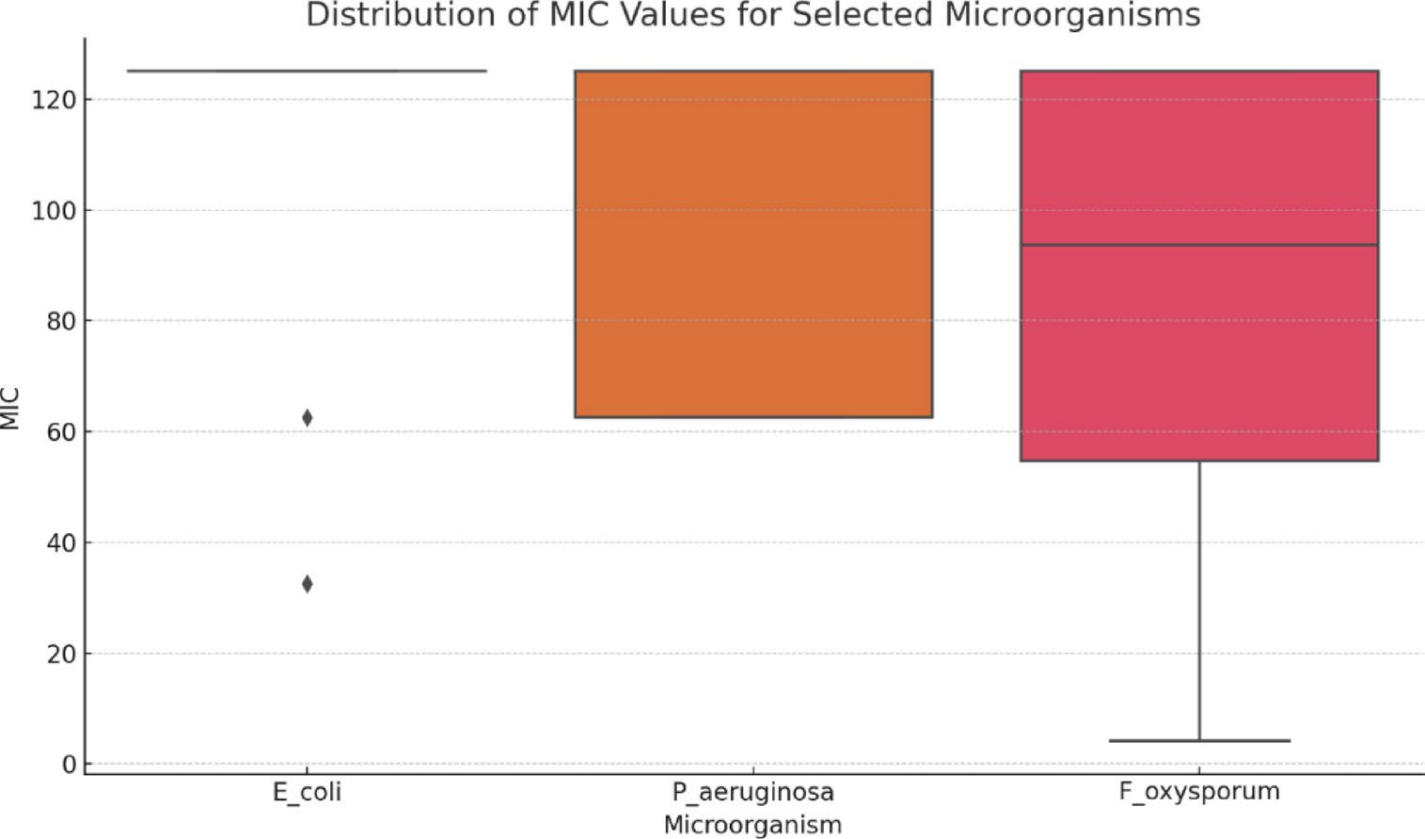
Distribution of MIC values for selected microorganisms.

**Boxplot: **Shows the distribution of MIC values for *E. coli*, *P. aeruginosa*, and *F. oxysporum*. Outliers like compound **4** (low MIC for *E. coli*) and compound **10** (low MIC for *F. oxysporum*) are visually prominent (Fig. 9).

**Correlation between MIC and inhibition zone**

**Fig. 10:**
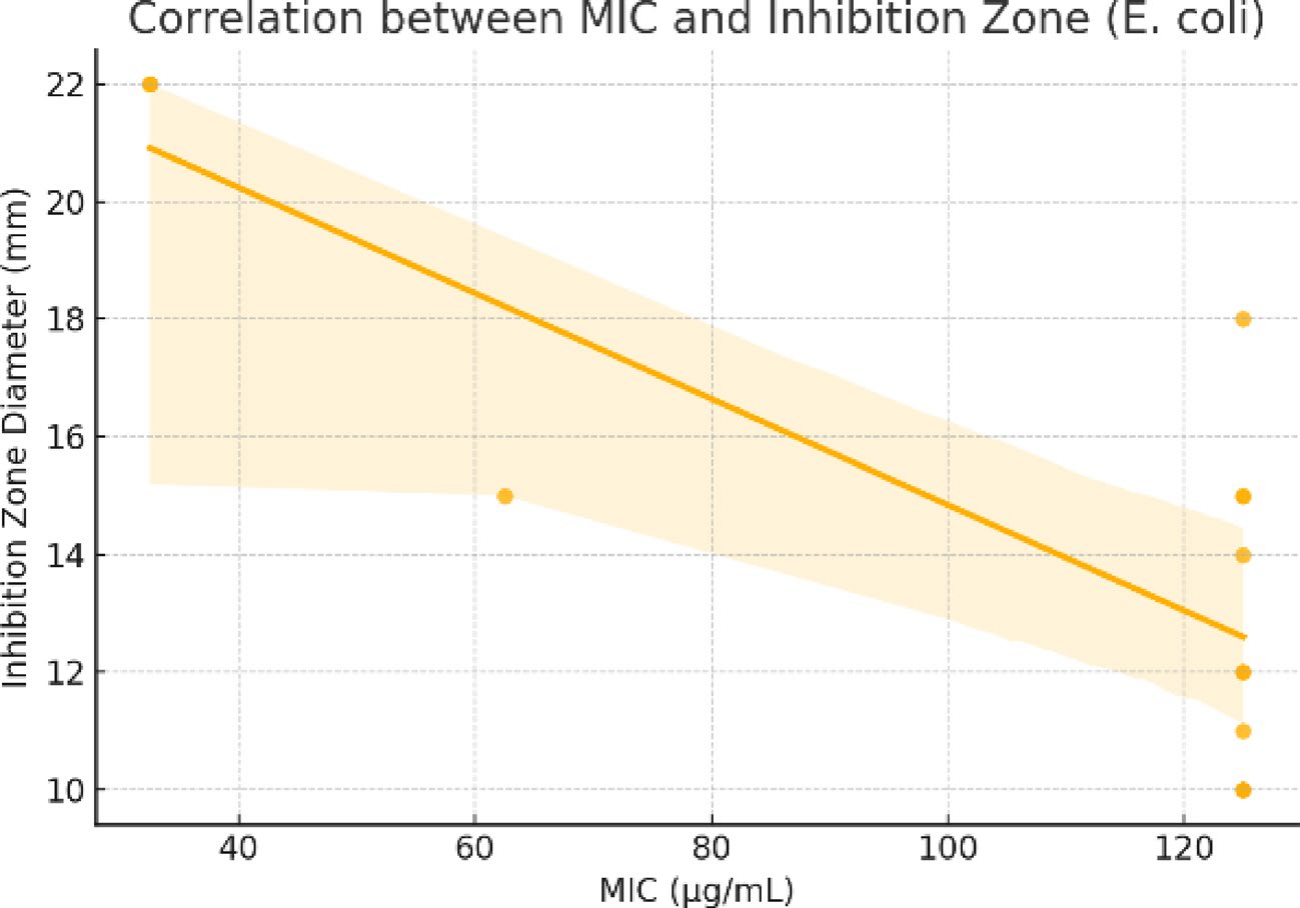
Correlation between MIC and inhibition zone (*E. coli*).

**Fig. 11:**
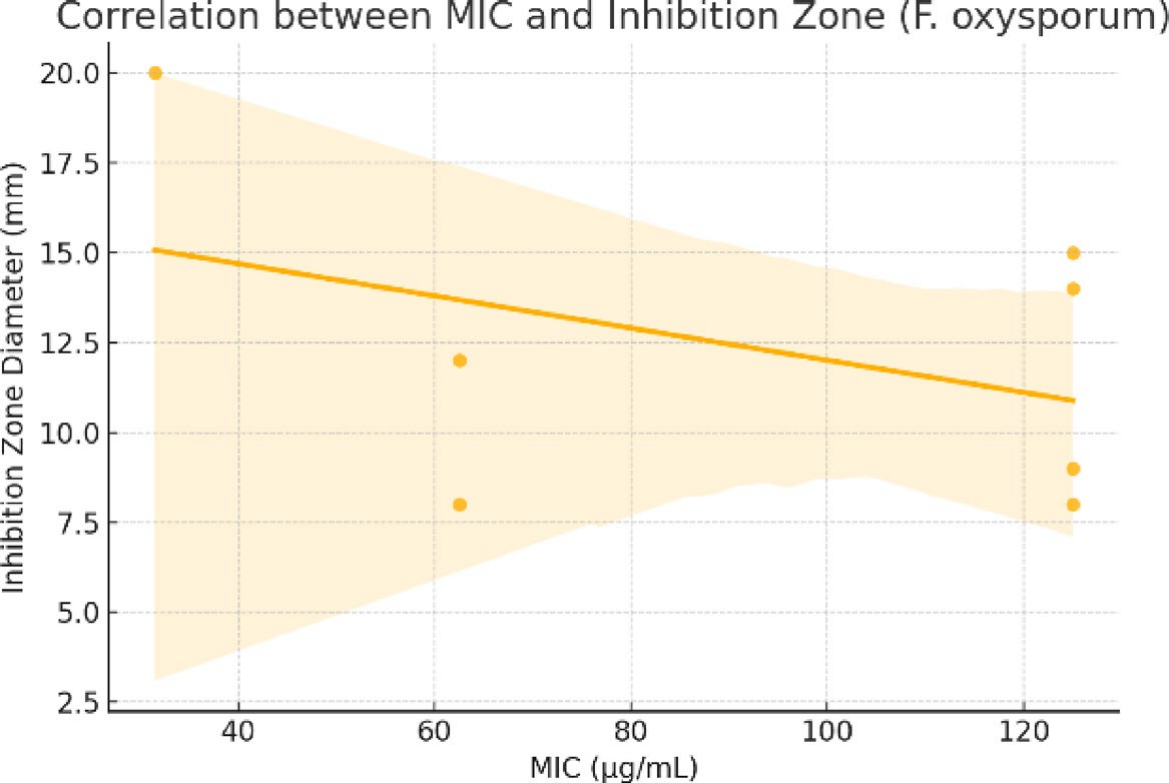
Correlation between MIC and inhibition zone (*F. oxysporum*).

**Fig. 12:**
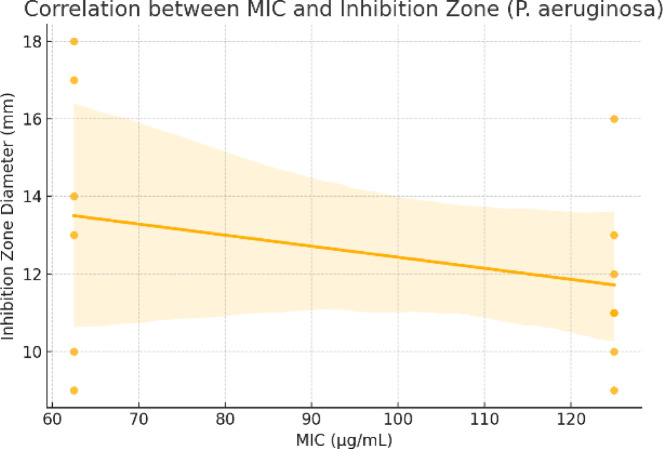
Correlation between MIC and inhibition zone (*P. aeruginosa*).

To validate the consistency between qualitative and quantitative antimicrobial assessments, a correlation analysis was performed between the minimum inhibitory concentrations (MICs) and the corresponding inhibition zone diameters for selected microorganisms. The results revealed a strong negative correlation for *E. coli* (r = − 0.79), indicating that compounds with lower MIC values generally produced larger zones of inhibition, which aligns with expected pharmacodynamic behavior. For *F. oxysporum*, the correlation was moderate (r = − 0.41), and for *P. aeruginosa*, it was weak (r = − 0.31) (Table 4). The weaker correlations observed for the fungal and Gram-negative strains may be attributed to differences in diffusion capacity, compound polarity, and cell wall permeability, which can affect zone sizes independently of MIC. These findings support the reliability of inhibition zone measurements for preliminary screening, but emphasize the necessity of MIC determination for accurate potency ranking, particularly when comparing structurally diverse compounds (Figs. 10, 11 and 12).

**Table 4:** Correlation Between MIC and inhibition zone.

Microorganism	Correlation coefficient (*r*)	Interpretation
*E. coli*	–0.79	Strong negative correlation
*F. oxysporum*	–0.41	Moderate negative correlation
*P. aeruginosa*	–0.31	Weak negative correlation

**Interpretation**

- A strong inverse correlation between MIC and inhibition zone was observed for *E. coli*. This means that compounds with lower MIC values tend to produce larger inhibition zones, as expected.
- The correlation is weaker for *F. oxysporum* and *P. aeruginosa*, possibly due to variability in compound diffusion in agar or different mechanisms of action and resistance in fungi and Gram-negative bacteria.

These relationships confirm that while inhibition zones offer a quick screening tool, MIC testing provides a more precise quantification of antimicrobial potency.

**Molecular docking**

**Fig. 13:**
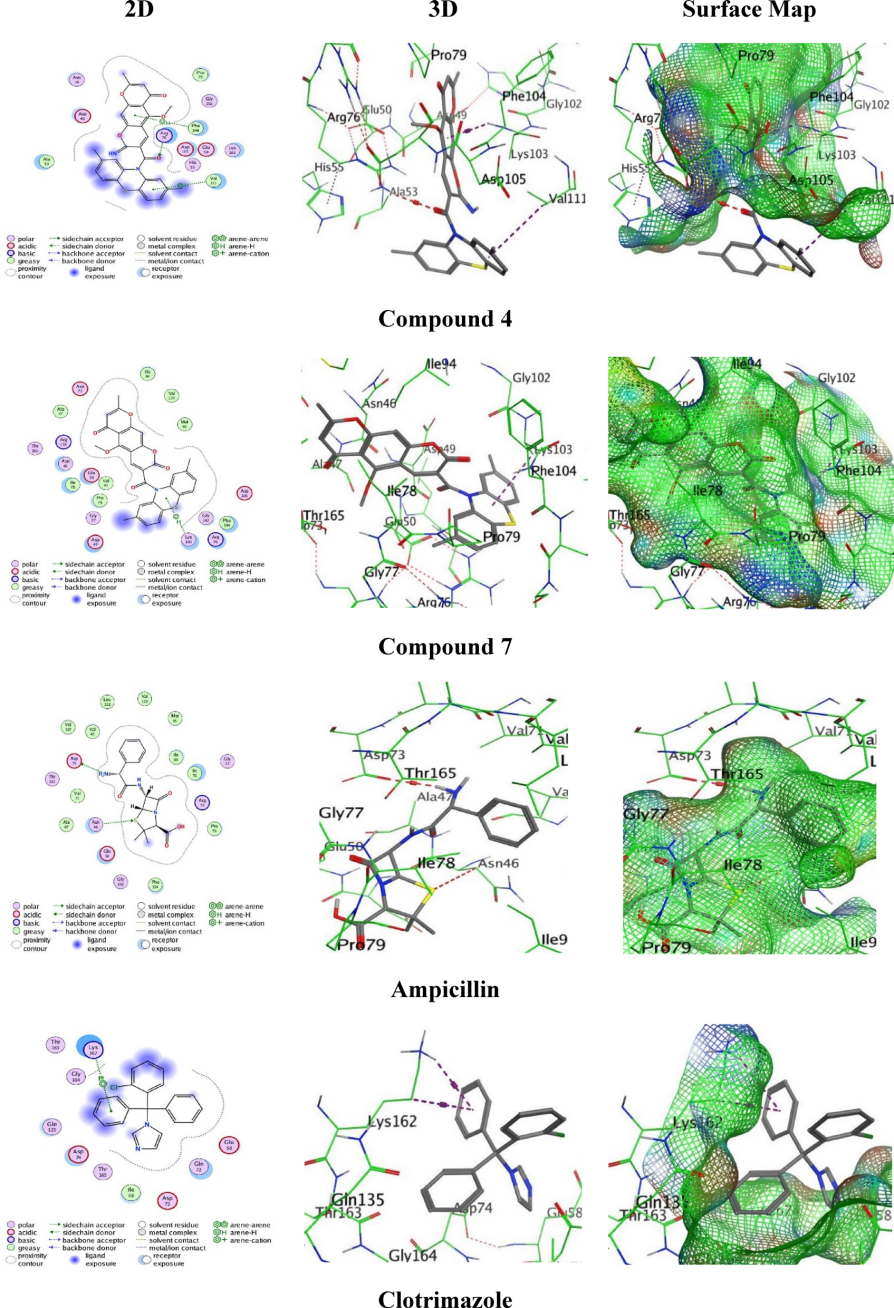
the docking images of the ligands 4, 7, Ampicillin, and Clotrimazole PDB: with 3G7E.

Molecular docking experiments revealed varied bindings and modes of the highest antibacterial compounds inside the *S. aureus β*-lactamase enzyme (PDB: 3G7B) (Fig. 13 and Table 5). The X-ray crystal structure of S. aureus β-lactamase, PDB ID: 3G7B, has been selected based on some criteria such as (1) high resolution at 1.80 Å to ensure atomic-level clarity for accurate ligand placement (2) the co-crystallized that captures a relevant ligand-bound conformation of the active site and one that is well-characterized and widely referenced for studying the inhibition of S. aureus β-lactamase to enable meaningful comparison of our results with existing computational and biochemical data (3) The use of this single high-quality structure provides a consistent and reliable framework for the comparative analysis of our synthesized compounds against the standard drugs. Compound **7** exhibited the stronger binding score (-5.9982 kcal/mol) with a repeatable root mean square (RMSD = 1.7480), creating a π-H interaction between its thiazine ring and Lys103 at an intermolecular distance = 4.20 Å. Meanwhile, compound **4** surveyed with a moderate binding score (S = -5.2067 kcal/mol through an RMSD of 1.4953), displayed various interactions: an H-acceptor bond between the amide oxygen (O 19) and Arg76 (3.41 Å), π-H stacking with Phe104 (4.20 Å), and a π-H stacking between the phenothiazine ring and Val111 (4.33 Å). However, Ampicillin (reference) showed a binding score (S = -5.8664 kcal/mol through RMSD = 0.9058), involving H-donor binding between its amino group and Asp73 (3.25 Å) and an H-acceptor bond with Asn46 through the sulfur (S 16) of its thiazole ring (3.93 Å). Moreover, clotrimazole (another standard) exhibited the lowest binding score (S = -5.0469 kcal/mol through an RMSD = 1.4232), forming double bindings between its unsubstituted benzene ring and Lys162 through both π-H (4.08 Å) and π-cation (4.22 Å) stacking. Conspicuously, compound **7** overtaken both reference drugs in binding energy, signifying improved stability and stronger bindings within the active site of the PDB: 3G7B protein.

**Table 5:** Molecular docking results between the potent compounds and PDB: 3G7E.

No.	Binding energy	RMSD	Ligands and amino acids interaction	Bond type	Distances (A°)
4	– 5.2067	1.4953	O 19 of the amide group with Arg76 Benzene-ring of chromone moiety with Phe104 Benzene-ring of phenothiazine moiety with Val111	H-acceptor pi-H pi-H	3.41 4.20 4.33
7	– 5.9982	1.7480	The thiazine-ring with Lys103	pi-H	4.20
Ampicillin	– 5.8664	0.9058	N 26 of the amino-group with Asp73 S 16 of the thiazole-ring with Asn46	H-donor H-acceptor	3.25 3.93
Clotrimazole	– 5.0469	1.4232	The unsubstituted benzene-ring with Lys162 The unsubstituted benzene-ring with Lys162	pi-H pi-cation	4.08 4.22

## Experimental

### Chemistry

#### Synthesis of 3-(2,8-dimethyl-10 H-phenothiazin-10-yl)-3-oxopropanenitrile (1)

2,8-dimethyl-10 H-Phenothiazine (10 mmol) was fused with an equimolar amount of cyanoacetic acid (10 mmol) in acetic anhydride (5 drops) at 80–85 °C in a boiling water bath for 40 min. Leave the reaction mixture for 1 h at room temperature, then filter off. The solid product was washed with ethanol (20 mL) to produce **2** as Off-white crystals.

Off-white crystals; Yield: 87% M.p.: 225–230 °C. IR (KBr): ν/cm^-1^=2229 (CN), 1681 (C = O); ^1^H-NMR (DMSO-d6) δ (ppm) = 2.17 (s, 6 H, 2 CH_3_), 3.032 (s, 2 H, CH_2_), 7.31–7.47 (m, 6 H, Ar-H); ^13^C-NMR (DMSO-d_6_): δ ppm 21.1, 22.8, 119.7, 126.1, 132.1, 133.1, 136.1, 138.1, 163.7; MS (EI, 70 eV): m/z (%) = 293 (M^+^-1; 49), 248 (79), 222 (52), 199 (100), 168 (55), 126 (68); Chemical Formula: C_17_H_14_N_2_OS (294.08); Calcd.: C, 69.36; H, 4.79; N, 9.52; O, 5.43; S, 10.89%; Found: C, 69.41; H, 4.72; N, 9.50; O, 5.39; S, 10.82%.

#### Synthesis of chromene-3-carboxamide derivatives 2, 3 and 4

**General procedure**:

A mixture of cyanoacetamide (**1**) (10 mmol) and salicylaldehyde, 2-hydroxy-1-naphthaldehyde, and 7-hydroxy-5-methoxy-2-methyl-4-oxo-*4 H*-chromene-6-carbaldehyde (10 mmol) in ethanol (30 ml) containing triethylamine (5 drops) as a base was heated under reflux for 0.5 h. The obtained solid product which formed while hot was collected by filtration and crystallized from ethanol: dioxane (2:1) to give **2–4** derivatives.

##### Synthesis of (2-imino-2 H-chromen-3-yl)(10 H-phenothiazin-10-yl) methanone (2)

light-yellow powder; Yield: 75% M.p.: 249–255 °C IR (KBr): ν/cm^− 1^=3210 − 3190 (NH), 1680 (C = O); ^1^H-NMR (DMSO-d_6_) *δ* (ppm) = 2.17 (s, 6 H, 2 CH_3_), 7.31–7.47 (m, 10 H, Ar-H), 8.03 (s, 1H, C_4_-H pyran), 9.49 (s, 1H, NH); ^13^C NMR (DMSO-d6): δ ppm 22.0, 39.5, 112.2, 114.0, 116.0, 120.0, 122.1, 129.5, 133.2, 136.1, 138.9, 154.0, 159.0, 160.9; MS (EI, 70 eV): *m/z* (%) = 398 (M^+^; 62), 258 (78), 198 (82), 167 (76), 97 (90); Chemical Formula: C_24_H_18_N_2_O_2_S (398.08); Calcd.: C, 72.34; H, 4.55; N, 7.03%; Found: C, 72.42; H, 4.52; N, 6.99%.

##### Synthesis of (2,8-dimethyl-10 H-phenothiazin-10-yl)(2-imino-2 H-benzo[h]chromen-3-yl)methanone (3)

Yellow powder; Yield: 70% M.p.: 252–255 °C; IR (KBr): ν/cm^− 1^=3218 (NH), 1682 (C = O); ^1^H-NMR (DMSO-d_6_) *δ* (ppm) = 2.29 (s, 6 H, 2CH3), 6.98–7.91 (m, 12 H, Ar-H), 8.66 (s, 1H, C_4_-pyran), 9.29 (s, 1H, NH); ^13^C NMR (DMSO-d6): δ ppm = 24.7, 26.7, 93.2, 110.5, 117.6, 122.0, 123.1, 128.0, 129.9, 136.6, 141.2, 143.4, 147.2, 155.2, 162.2, 167.9, 172.9 197.1; MS (EI, 70 eV): *m/z* (%) = 448 (M^+^; 28), 162 (78), 90 (40); Chemical Formula: C_28_H_20_N_2_O_2_S : (448.08); Calcd.: C, 74.98; H, 4.49; N, 6.25%; Found: C, 74.96; H, 4.40; N, 6.28%.

##### 2-imino-5-methoxy-8-methyl-3-(10 H-phenothiazine-10-carbonyl)-2 H,6 H-pyrano[3,2 g] chromen-6-one (4)

Beige powder; Yield: 78% M.p.: 260–265 °C ; IR (KBr): ν/cm^− 1^=3186 (NH), 1700, 1650 (2 C = O); ^1^H-NMR (DMSO-d_6_) *δ* (ppm) = 2.41 (s, 6 H, 2 CH_3_), 2.44 (s, 3 H, CH_3_), 3.83 (s, 3 H, O-CH_3_), 7.47–7.87 (m, 8 H, Ar-H), 8.60 (s, 1H, C_4_-pyran), 9.85 (s, 1H, NH); ^13^C NMR (DMSO-d6): δ ppm = 19.5, 21.0, 62.4, 97.3, 105.5, 110.2, 112.4, 116.0, 119.1, 128.1, 132.7, 135.6, 138.0, 159.1, 162.6, 165.0, 175.9; MS (EI, 70 eV): *m/z* (%) = 510 (M^+^; 65), 484 (40), 275 (35), 176 (44), 108 (100); Chemical Formula: C_29_H_22_N_2_O_5_S : (510.08); Calcd.: C, 68.22, H, 4.34, N, 5.49%; Found: C, 68.19, H, 4.30, N, 5.50%.

#### Synthesis of substituted coumarin derivatives 5, 6 and 7

**General method**:

Compounds **2**,** 3**, and **4** (0.01 mol) were heated in a mixture of conc. HCl and ethanol (1:1, 20 ml) for 30 min. The reaction mixture was left to stand at room temperature overnight and the solid product was filtered off, dried, and recrystallized from ethanol to give corresponding chromenone derivatives **5**,** 6**, and **7**.

##### Synthesis of 3-(10 H-phenothiazine-10-carbonyl)-2 H-chromen-2-one (5)

Yellow crystals; yield 69% m.p. 196 °C; IR (KBr): ν/cm^− 1^=1695, 1648 (2 C = O); ^1^H-NMR (DMSO-d_6_) *δ* (ppm) = 2.43 (s, 6 H, 2CH_3_), 7.31–7.86 (m, 10 H, Ar-H), 8.46 (s, 1H, C_4_-pyranone); ^13^C NMR (DMSO-d6): δ ppm = 24.7, 26.7, 93.2, 110.5, 117.6, 122.0, 123.1, 128.0, 129.9, 136.6, 141.2, 143.4, 147.2, 155.2, 162.2, 167.9, 172.9 197.1; MS (EI, 70 eV): *m/z* (%) = 399 (M^+^; 70), 198 (65), 157 (32), 98(100); Chemical Formula: C_24_H_17_NO_3_S : (399.06), Calcd.: C, 72.16, H, 4.29, N, 3.51%; Found: C, 72.10, H, 4.20, N, 3.50%.

##### Synthesis of 3-(2,8-dimethyl-10 H-phenothiazine-10-carbonyl)-2 H-benzo[h]chromen-2-one (6)

Brown crystals; yield 58% m.p. 175 °C; IR (KBr): ν/cm^− 1^=1715, 1655 (2 C = O); ^1^H-NMR (DMSO-d_6_) *δ* (ppm) = 2.49 (s, 6 H, CH_3_), 7.30–8.19 (m, 12 H, Ar-H), 8.39 (s, 1H, C_4_-pyran); ^13^C NMR (DMSO-d6): δ ppm = 21.9, 114.2, 115.9, 117.1, 118.5, 122.6, 126.1, 130.5, 132.5, 135.3, 137.6, 144.9, 150.2, 159.5; MS (EI, 70 eV): *m/z* (%) = 450 (M^+^+1; 40); Chemical Formula: C_28_H_19_NO_3_S : (449.08). Calcd.: C, 74.81, H, 4.26, N, 3.12%, Found: C, 74.79, H, 4.23, N, 3.09%.

##### Synthesis of 5-methoxy-8-methyl-3-(10 H-phenothiazine-10-carbonyl)-2 H,6 H-pyrano [3,2-g]chromene-2,6-dione (7)

Orange crystals; yield 58% m.p. 175 °C; IR (KBr): ν/cm^− 1^=1730, 1685 and 1645 (3 C = O); ^1^H-NMR (DMSO-d_6_) *δ* (ppm) = 2.30 (s, 6 H, 2CH_3_), 2.43 (s, 3 H, CH_3_), 3.79 (s, 3 H, OCH_3_), 6.29 (s, 1H, C_7_-H pyranone), 6.46–7.11 (m, 6 H, Ar-H); ^13^C NMR (DMSO-d6): δ ppm = 24.7, 26.7, 93.2, 110.5, 117.6, 122.0, 123.1, 128.0, 129.9, 136.6, 141.2, 143.4, 147.2, 155.2, 162.2, 167.9, 172.9 197.1; MS (EI, 70 eV): *m/z* (%) = 511 (M^+^; 73), 482 (80), 315 (79), 197(75), 111 (70), 77 (100); Chemical Formula: C_29_H_21_NO_6_S : (511.08): Calcd.: C, 68.09, H, 4.14, N, 2.74%; Found: C, 68.10, H, 4.12, N, 2.79%.

#### Synthesis of phenothiazine derivatives 8a, b

**General method**

To a solution of **1** (10 mmol) in DMF (25 mL) containing a catalytic amount of pyridine (1.00 mL), either benzaldehyde(10 mmol) or acetaldehyde (10 mmol) was added. The reaction mixture, in each case, was heated under reflux for 5 h. the solid products formed upon pouring onto an ice-water mixture containing a few drops of hydrochloric acid to yield the benzylidene derivatives **8a**,** b**.

##### Synthesis of 2-(10 H-phenothiazine-10-carbonyl)-3-phenylacrylonitrile (8a)

White crystals; yield 64% m.p. 187 °C; IR (KBr): ν/cm^− 1^=2218 (CN), 1680 (C = O); ^1^H-NMR (DMSO-d_6_) *δ* (ppm) = 2.40 (s, 6 H, 2 CH_3_), 7.31–7.47 (m, 11 H, Ar-H), 8.17 (s, 1H, CH); ^13^C NMR (DMSO-d6): δ ppm = 24.7, 26.7, 93.2, 110.5, 117.6, 122.0, 123.1, 128.0, 129.9, 136.6, 141.2, 143.4, 147.2, 155.2, 162.2, 167.9, 172.9 197.1; MS (EI, 70 eV): *m/z* (%) = 382 (M^+^; 55); Chemical Formula: C_24_H_18_N_2_OS : (382.08); Calcd.: C, 75.37, H, 4.74, N, 7.32%; Found: C, 75.32, H, 4.70, N, 7.34%.

##### Synthesis of 2-(10 H-phenothiazine-10-carbonyl)-3-phenylbut-2-enenitrile (8b)

Off white crystals; yield 45% m.p. 221 °C; IR (KBr): ν/cm^− 1^=2220 (CN), 1675 (C = O); ^1^H-NMR (DMSO-d_6_) *δ* (ppm) = 2.21 (s, 6 H, 2CH_3_), 2.41 (s, 3 H, CH_3_), 6.83–7.71 (m, 11 H, Ar-H); ^13^C NMR (DMSO-d6): δ ppm = 13.8, 21.5, 95.9, 115.9, 119.5, 126.9, 128.6, 132.5, 135.7, 138.1, 140.2, 158.9, 174.1; MS (EI, 70 eV): *m/z* (%) = 396 (M^+^; 40), 303 (48), 199 (100), 123(80); Chemical Formula: C_25_H_20_N_2_OS : (396.10); Calcd.: C, 75.73, H, 5.08, N, 7.07%; Found: C, 75.70, H, 5.11, N, 7.09%.

#### Synthesis of (2-imino-4,6-dimethyl-2 H-pyran-3-yl)(10 H-phenothiazin-10-yl)methanone (9)

A solution of compound **1** (10 mmol), in DMF (30 mL), in the presence of a catalytic amount of triethylamine (4 drops), and acetylacetone (10 mmol) was refluxed 5–7 h. The reaction mixture was allowed to cool and poured onto crushed ice. The obtained solid product was collected by filtration, washed, dried, and crystallized from ethanol to give compound **9.**

Gray powder; yield (70%); m.p. >300°**C**; IR (KBr): ν/cm^− 1^= 3219 (NH), 1685 (C = O); ^1^H-NMR (DMSO-d_6_) *δ* (ppm) = 1.80 (s, 1H, CH_3_), 2.37 (s, 6 H, 2CH_3_ ), 2.55 (s, 3 H, CH_3_), 7.40 (s, 1H, C_3_-H pyran), 7.70–7.75 (m, 6 H, Ar-H), 9.50 (s, 1H, NH); ^13^C NMR (DMSO-d6): δ ppm = 19.4, 20.0, 22.8, 106.0, 113.4, 123.4, 133.1, 135.0, 139.3, 145.0, 158.4, 162.2, 197.4; MS (EI, 70 eV): *m/z* (%) = 376 (M^+^; 38), 258 (48), 172 (59); Chemical Formula: C_22_H_20_N_2_O_2_S : (376.09); Calcd.: C, 70.19, H, 5.35, N, 7.44%; Found: C, 70.20, H, 5.30, N, 7.42%.

#### Synthesis of (4-amino-3-phenyl-2-thioxo-2,3-dihydrothiazol-5-yl)(10 H-phenothiazin-10-yl)methanone (11)

To a solution of **1** (10 mmol) in DMF (25mL) containing a catalytic amount of triethylamine (1.00 mL) and phenyl isothiocyanate (10 mmol) was added followed by the addition of an equimolar amount of elemental sulfur (10 mmol). The reaction mixture was heated under reflux for 5 h, then cooled and neutralized by pouring onto an ice-water mixture containing a few drops of hydrochloric acid. The solid product formed was collected by filtration and crystallized from DMF.

Yellow crystals; yield (72%); m.p. 185 °C; IR (KBr): ν/cm^− 1^= 3399 (NH_2_), 1720 (C = O), 1310 (C = S); ^1^H-NMR (DMSO-d_6_) *δ* (ppm) = 2.49 (s, 6 H, 2 CH_3_), 6.44 (s, 2 H, NH_2_), 7.31–7.47 (m, 11 H, Ar-H); ^13^C NMR (DMSO-d6): δ ppm = 21.0, 75.5, 119.9, 129.0, 133.0, 134.0, 136.0, 138.7, 159.9, 184.8; MS (EI, 70 eV): *m/z* (%) = 462 (M^+^ +1, 35), 339 (60), 264 (62), 199 (100), 126 (68); Chemical Formula: C_24_H_19_N_3_OS_3_ : (461.04); Calcd.: C, 62.44, H, 4.15, N, 9.10%; Found: C, 62.46, H, 4.18, N, 9.12%.

#### Synthesis of 2,4-diamino-5-(10 H-phenothiazine-10-carbonyl)thiophene-3-carbonitrile (13)

A solution of **1** (10 mmol) in DMF (25mL) containing a catalytic amount of triethylamine (1.00 mL) and malononitrile (10 mmol) was added followed by the addition of an equimolar amount of elemental sulfur (10 mmol). The reaction mixture was heated under reflux for 5 h, then cooled and neutralized by pouring onto an ice-water mixture containing a few drops of hydrochloric acid. The solid product formed was collected by filtration and crystallized from DMF.

Orange crystals; yield (78%); m.p. 198°**C**; IR (KBr): ν/cm^− 1^= 3420, 3398 (2NH_2_), 2218 (CN), 1682 (C = O); ^1^H-NMR (DMSO-d_6_) *δ* (ppm) = 2.47 (s, 6 H, 2 CH_3_), 6.60 (s, 2 H, NH_2_), 6.66–7.11 (m, 8 H, Ar-H + NH_2_); ^13^C NMR (DMSO-d6): δ ppm = 21.5, 82.4, 111.4, 115.4, 123.4, 126.8, 128.0, 133.1, 139.37, 158.4, 162.2; MS (EI, 70 eV): *m/z* (%) = 392 (M^+^; 50), 294 (82), 217 (40), 77(100); Chemical Formula: C_20_H_16_N_4_OS_2_ : (392.05); Calcd.: C, 61.20, H, 4.11, N, 14.27%; Found: C, 61.17, H, 4.15, N, 14.31%.

#### Synthesis of (3-amino-5-phenyl-1 H-pyrazol-4-yl)(10 H-phenothiazin-10-yl)methanone (14)

To a solution of **8a** (10 mmol) in DMF (25mL) hydrazine hydrate (10 mmol) was added. The reaction mixture was heated under reflux for 5 h. the solid product formed upon pouring onto an ice-water mixture containing a few drops of hydrochloric acid was collected by filtration and recrystallized from DMF to produce compound 14.

White crystals; yield 60% m.p. 248 °C; IR (KBr): ν/cm^− 1^= 3420 (NH_2_), 3204 (NH), 1705 (C = O); ^1^H-NMR (DMSO-d_6_) *δ* (ppm) = 2.28 (s, 6 H, 2 CH_3_), 6.20 (s, 2 H, NH_2_), 6.96–7.67 (m, 11 H, Ar-H), 9.02 (s, 1H, NH); ^13^C NMR (DMSO-d6): δ ppm = 21.7, 96.6, 119.4,125.9, 127.7, 129.5, 131.7, 136.0, 137.0, 154.8, 156.9; MS (EI, 70 eV): *m/z* (%) = 412 (M^+^; 28), 377 (33), 256 (28), 199 (100), 69 (88); Chemical Formula: C_24_H_20_N_4_OS : (412.10); Calcd.: C, 69.88, H, 4.89, N, 13.58%; Found: C, 69.90, H, 4.86, N, 13.60%.

### Antimicrobial evaluation^24^

The antimicrobial activity of the synthesized compounds was evaluated against a panel of microorganisms comprising Gram-negative bacteria (*Escherichia coli*, *Pseudomonas aeruginosa*), **Gram-positive bacteria** (*Staphylococcus aureus*, *Bacillus subtilis*), and fungi (*Candida albicans* and *Fusarium oxysporum*). The strains were obtained from the Microbiology Laboratory of [Institution/Lab name], where they are maintained as stock cultures on appropriate agar media and sub-cultured prior to testing.

#### Inoculum preparation

Bacterial strains were grown overnight on Mueller–Hinton agar (MHA) at 37 °C. Several colonies were suspended in sterile saline (0.85% NaCl) and adjusted spectrophotometrically to the turbidity of a 0.5 McFarland standard (≈ 1.0 × 10⁸ CFU/mL). This suspension was then diluted 1:100 in Mueller–Hinton broth (MHB) to obtain a final inoculum of approximately 1.0 × 10⁵ CFU/mL for MIC determination.

*Candida albicans* was grown on Sabouraud dextrose agar (SDA) at 30 °C for 24 h, and *F. oxysporum* on potato dextrose agar (PDA) at 28–30 °C for 48–72 h. Yeast and fungal spores were harvested in sterile saline containing 0.05% Tween-80, filtered to remove mycelial fragments (for *F. oxysporum*), and adjusted to ~ 10⁵ CFU/mL (or spores/mL) using a hemocytometer.

#### MIC determination (broth microdilution assay)

Minimum inhibitory concentrations (MICs) were determined using a standard broth microdilution method in sterile 96-well microplates, adapted from Fadda et al.^24^. Stock solutions of the test compounds were prepared in DMSO and further diluted with MHB (for bacteria) or Sabouraud dextrose broth (SDB, for fungi) so that the final DMSO concentration in wells did not exceed 1% v/v, which had no effect on microbial growth.

Two-fold serial dilutions of each compound were prepared in the microplates to give a final concentration range of, for example, 3.9–250 µg/mL. Each well was inoculated with 100 µL of the standardized microbial suspension to reach a final inoculum of ~ 10⁵ CFU/mL. For each plate, the following controls were included:

- **Sterility control**: medium only (no inoculum, no compound).
- **Growth control**: inoculated medium without test compound.
- **Solvent control**: inoculated medium containing the maximum DMSO concentration used.
- **Positive controls**: ampicillin for bacterial strains and clotrimazole for fungal strains, tested over the same concentration range as reference drugs.

Microplates were incubated at 37 °C for 24 h for bacteria, 0 °C for 24 h for *C. albicans*, and 28–30 °C for 48–72 h for *F. oxysporum*. After incubation, wells were visually inspected for turbidity. Whenever necessary, optical density at 600 nm was measured using a microplate reader to confirm growth inhibition.

The MIC was defined as the lowest concentration of compound that completely inhibited visible growth (no turbidity compared with the sterility control). For fungi, MIC was similarly taken as the lowest concentration preventing visible growth of yeast cells or mycelial development.

#### Inhibition zone assay

For correlation analysis between inhibition zones and MIC values, an agar diffusion (disc-diffusion) assay was also performed. Sterile 6-mm paper discs were impregnated with a fixed amount of each compound ([xx] µg/disc; adjust as used) and placed on MHA (for bacteria) or SDA/PDA (for fungi) plates previously inoculated with standardized microbial suspensions (0.5 McFarland). Plates were incubated under the same conditions described above, and the diameters of inhibition zones (mm) were measured in two perpendicular directions and averaged.

#### Replicates and data analysis

All antimicrobial experiments (MIC determination and inhibition zone measurements) were performed in triplicate in three independent experiments (n = 3). MIC values in Table 2 are expressed as mean ± standard error of the mean (SEM), and the SEM was used to generate error bars in the corresponding figures. Statistical comparisons among compounds were carried out as described in the *Statistical Analysis* section, using one-way ANOVA followed by Tukey’s post hoc test, with *p* < 0.05 considered statistically significant.

### Molecular docking

Protein Data (https://www.rcsb.org) provided the X-ray crystal structure of S. aureus β-lactamase enzyme (PDB ID: 3G7B)^25^. Though, the chemical structures of compounds **4**, **7**, and the drug references were created using the (ChemDraw 16.0 professional) application with appropriate 2D, 3D, and surface Map alignment, and each molecule’s energy was decreased using ChemBio3D. In the meantime, the target protein was saved in PDB format before being exported into the M.O.E. program, which generated the ligands and saved them in PDB format. The water molecules and the native co-crystallized ligand were removed, hydrogen atoms were added, and the protein structure was prepared by assigning partial charges using the AMBER10:EHT force field. In accordance to the docking validation, the selected ligand was re-docked into the distinct active site. However, an RMSD value below 2.0 Å confirms the reliability of the docking parameters. Rendering to this re-docked ligand, the binding site was well-defined covering all the key active site residues of β-lactamase, including Ser70, Ser130, Lys73, Lys234, and Asn132, which are reported as crucial for its catalytic activity.

## Statistical analysis

To assess the antimicrobial efficacy of the synthesized compounds, statistical analysis was conducted using a combination of descriptive and inferential methods. Minimum Inhibitory Concentration (MIC) values were analyzed to calculate the mean, standard deviation, range, and number of active strains for each compound, allowing for a comparative evaluation of potency^26^. One-way ANOVA was performed to determine statistically significant differences in MIC values among compounds for each microorganism, with a significance level set at *p* < 0.05^27^. Where significant differences were found, Tukey’s post hoc test was applied to identify specific compound pairs with differential activity^28^. Hierarchical clustering was used to group compounds with similar MIC profiles, highlighting structural activity relationships^29^. Visual tools such as heatmaps, boxplots, and dendrograms were employed to illustrate trends in antimicrobial potency. Additionally, correlation analysis between MIC values and inhibition zone diameters was conducted for selected organisms, revealing a strong negative correlation for *E. coli* (*r* = − 0.79), moderate for *F. oxysporum* (*r* = − 0.41), and weak for *P. aeruginosa* (*r* = − 0.31), indicating that while inhibition zones are useful for preliminary screening, MIC values provide a more accurate measure of antimicrobial activity^30^.

**Conclusion**

In this study, a novel series of phenothiazine-based heterocyclic compounds were successfully synthesized and structurally characterized. The incorporation of chromene, cyanoacetamide, and various heteroaromatic moieties enhanced the antimicrobial potential of the phenothiazine scaffold. Several synthesized compounds, particularly compounds 4, 7, and 10, demonstrated significant antimicrobial activity against both bacterial and fungal strains, with compound 10 showing the most potent antifungal activity. Drug-likeness evaluation using Lipinski’s rule of five revealed favorable pharmacokinetic profiles for most derivatives. Moreover, molecular docking studies confirmed strong binding affinities of compounds 4 and 7 to the β-lactamase active site, with compound 7 surpassing the binding efficiency of reference drugs. The combined synthetic, biological, and computational results highlight these phenothiazine hybrids as promising candidates for further development as broad-spectrum antimicrobial agents, especially in the context of combating multidrug-resistant pathogens.

## Conclusion

In this report, some new phenothiazine- based heterocyclic compounds were successfully synthesized and comprehensively characterized. Antimicrobial evaluation showed that most the tested compounds exhibited remarkable activity; compounds 4, 7, and 10 were highly active against bacteria and fungi. Additionally, drug-likeness calculations revealed that most of the synthesized compounds conformed to Lipinski’s rule, thereby possessing suitable physicochemical and pharmacokinetic profiles for oral administration. Furthermore, molecular docking studies supported their biological activities, as compound 7 showed the highest binding affinity toward the β-lactamase enzyme (PDB: 3G7B), with a higher predicted interaction energy and stability than ampicillin and clotrimazole. Statistical analyses confirmed these activity differences to be highly significant and pointed out strong correlations between MIC values and inhibition zones for key strains. All these synthetic, biological, computational, and statistical aspects establish compounds 4, 7, and 10 as promising leads toward the synthesis of next-generation antimicrobial agents. Their effectiveness against both Gram-positive and Gram-negative bacteria, along with encouraging antifungal activities and favorable drug-likeness, makes them valuable scaffolds for future optimization in the quest for active drugs against multidrug-resistant pathogens.

## Supplementary Information

Below is the link to the electronic supplementary material.

Supplementary Material 1

## Data Availability

The datasets generated and/or analysed during the current study are available in this published article and submitted as a supplementary file.
