# Supplementary material for: Synthesis, antimicrobial evaluation, molecular docking, and drug-likeness assessment of novel phenothiazine chromene hybrid compounds
Source: Sci Rep. 2026 Mar 30;16:10592. doi: 10.1038/s41598-026-43195-3 (PMC13039439; doi:10.1038/s41598-026-43195-3)
Supplement: Supplementary file 1 — Supplementary Material 1 [file 41598_2026_43195_MOESM1_ESM.docx]

Supplementary Data for the Synthesized Compounds


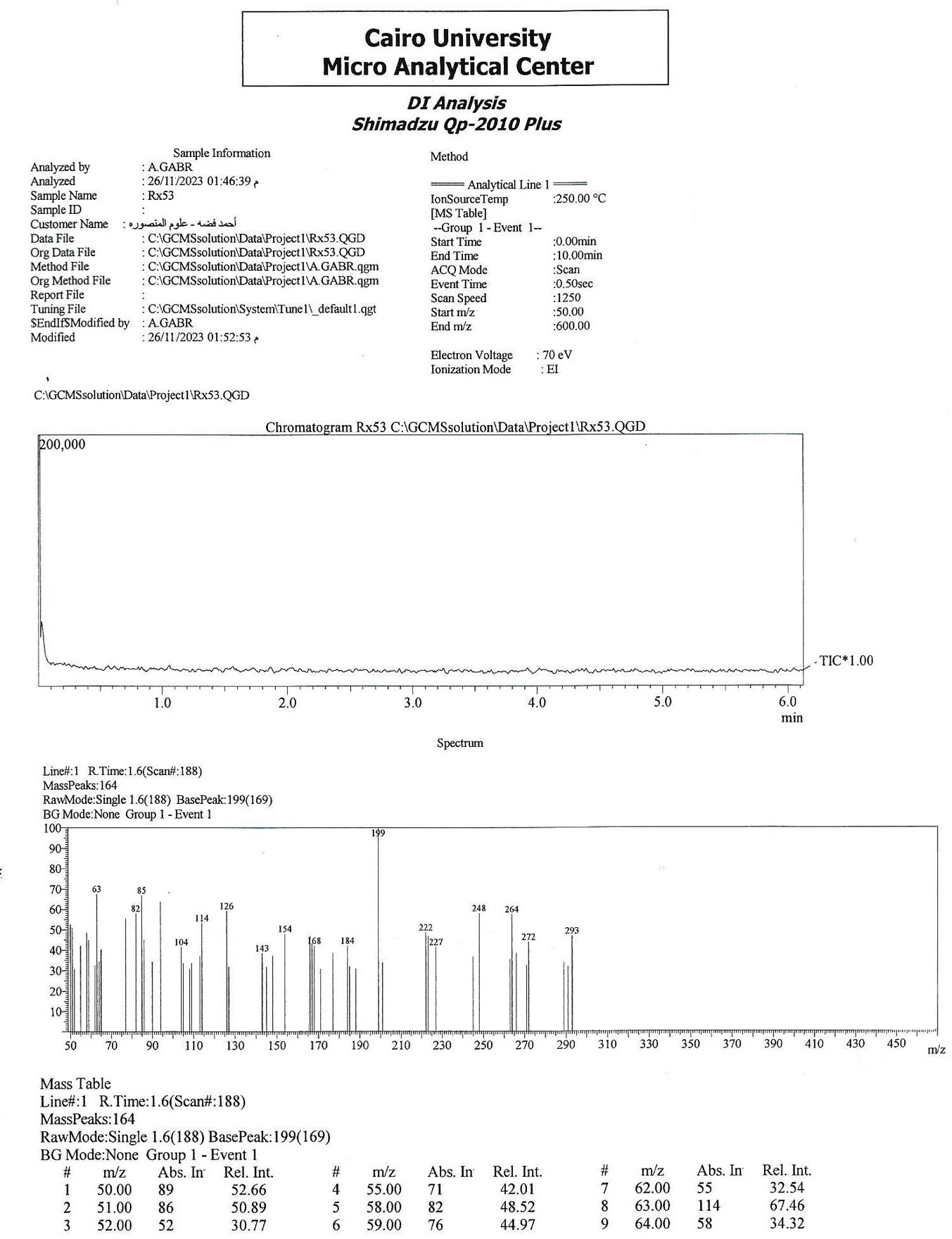


Compound 1 Mass spectrum


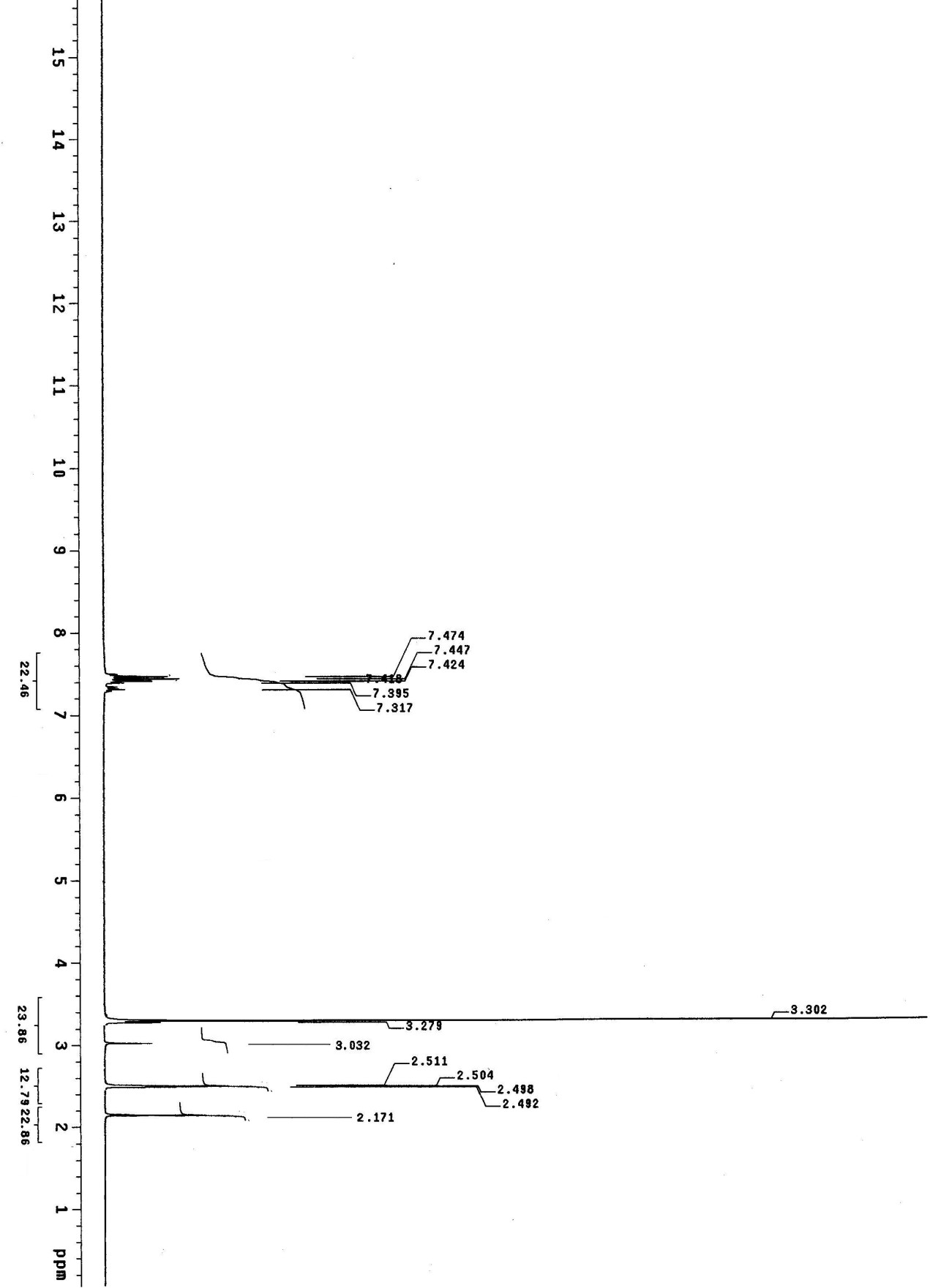


Compound 1 ^1^H-NMR


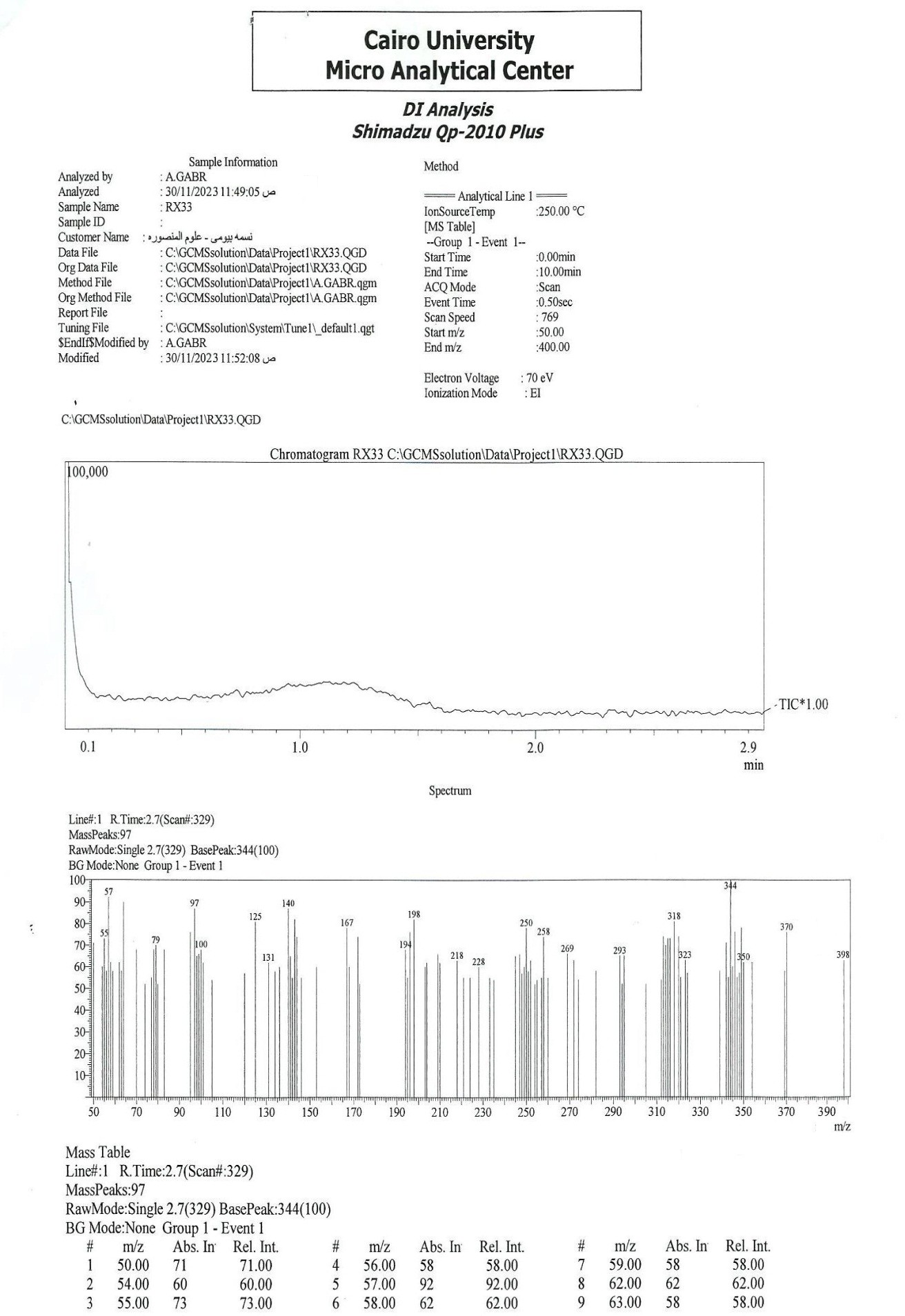


Compound 2 Mass spectrum


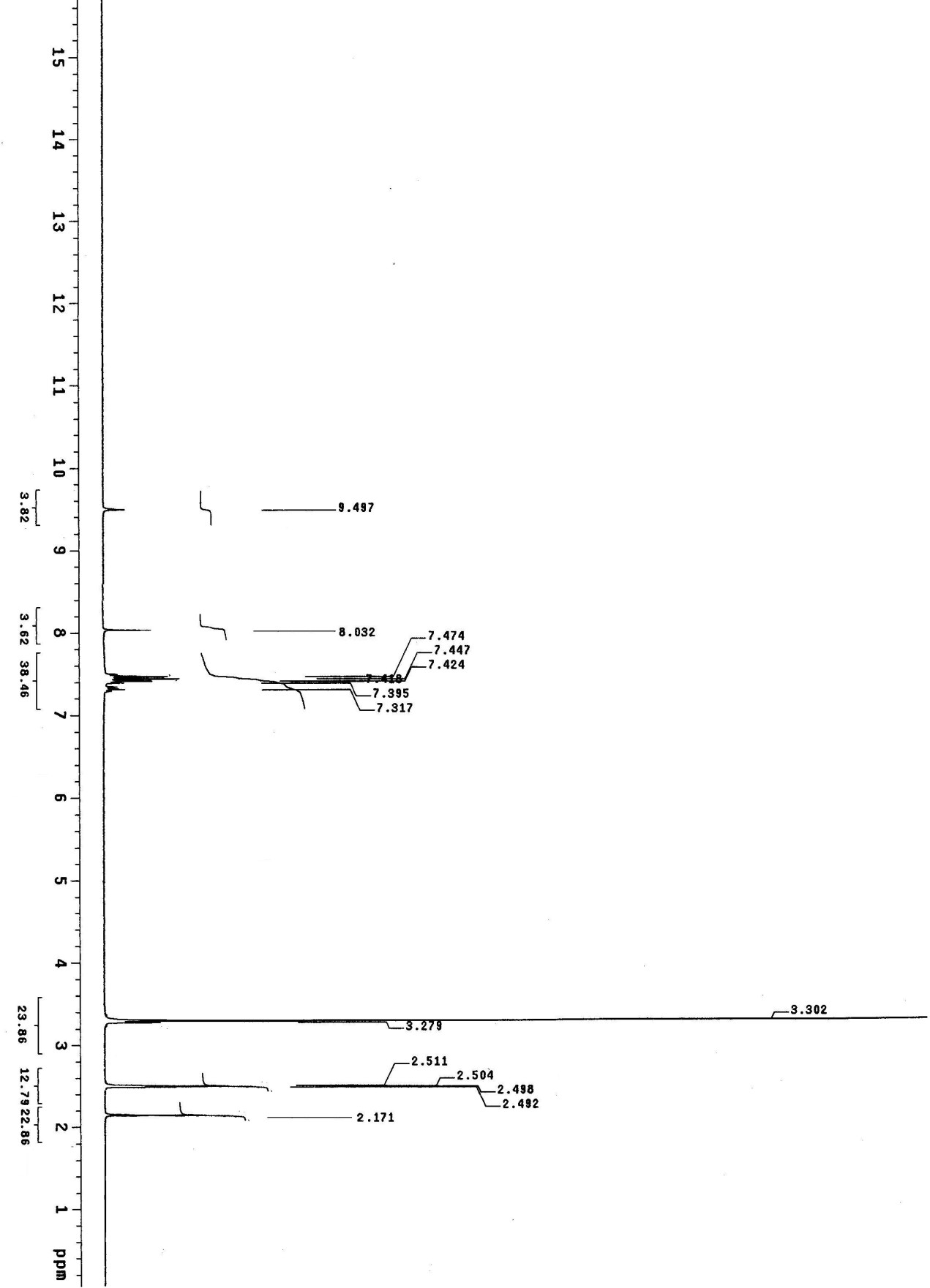


Compound 2 ^1^H-NMR


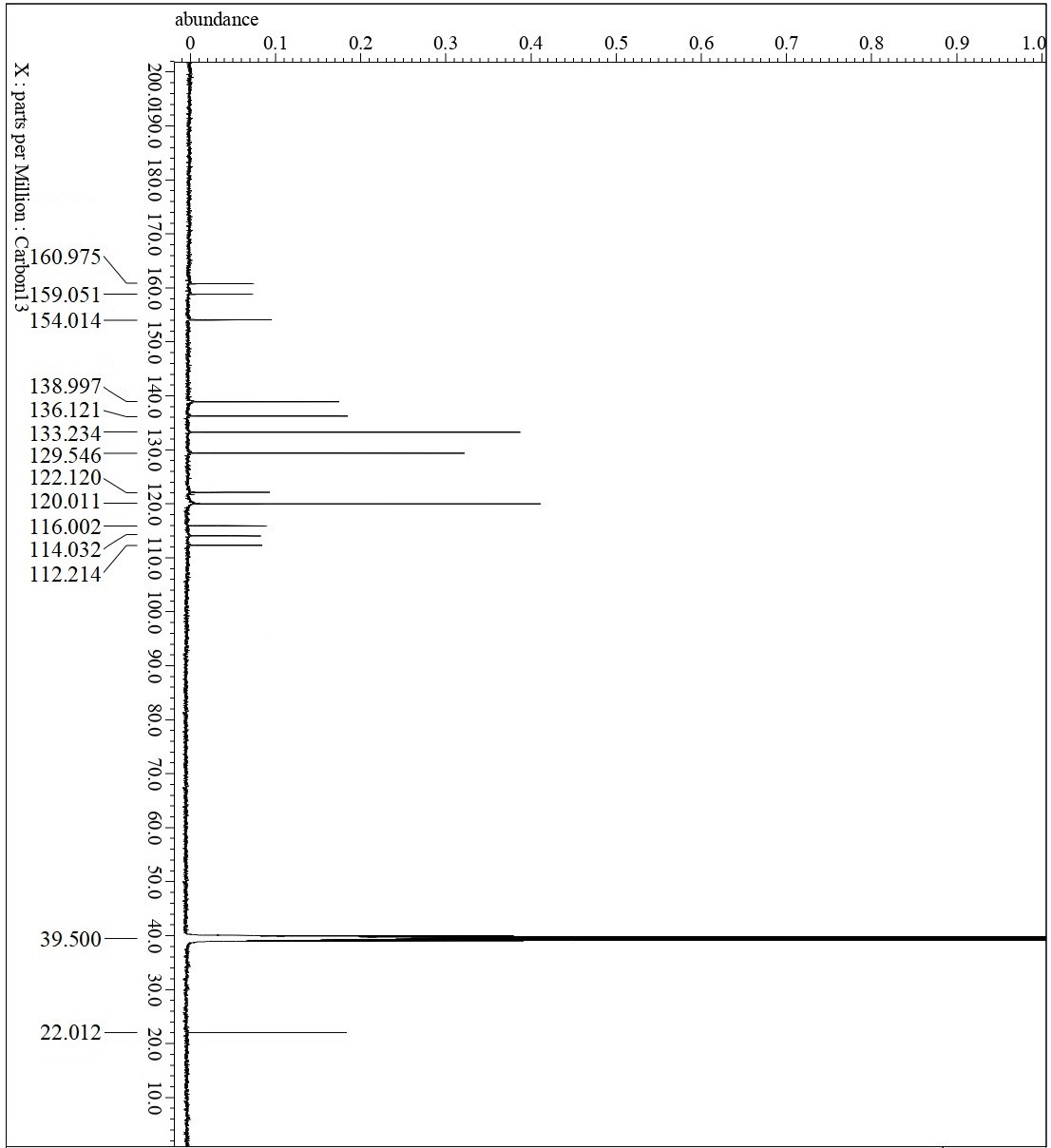


Compound 2 ^13^C-NMR


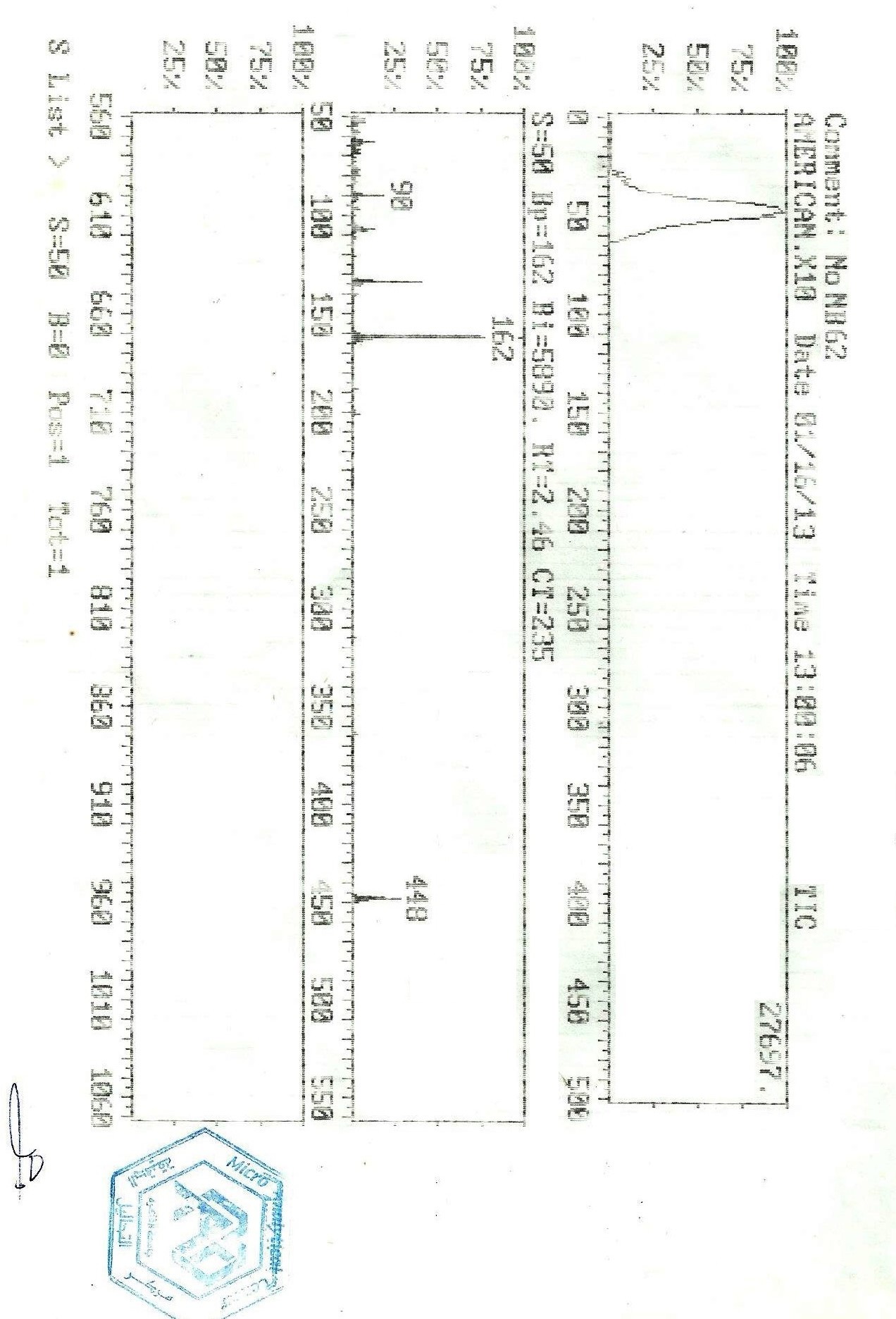


Compound 3 Mass spectrum


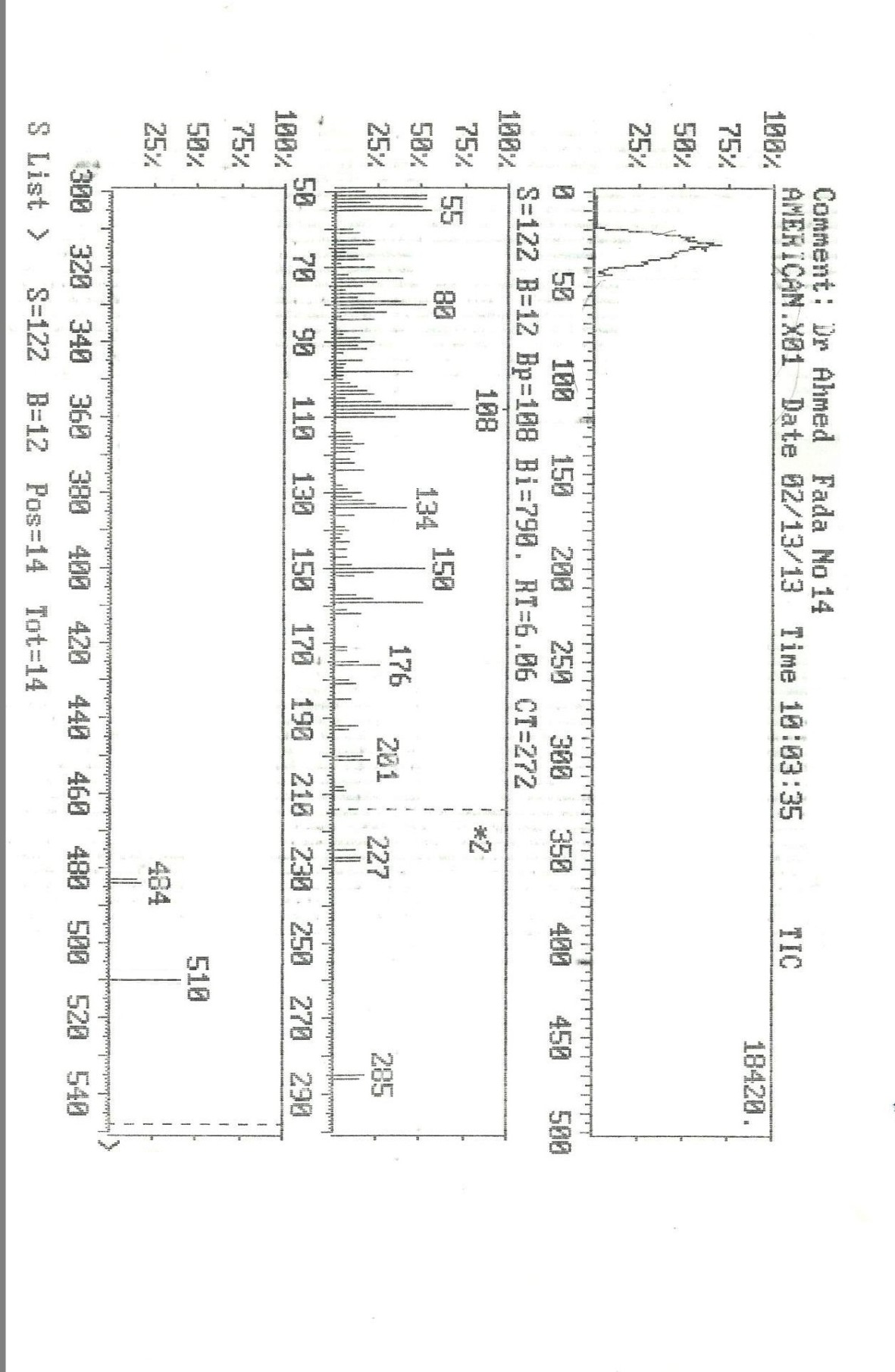


Compound 4 Mass spectrum


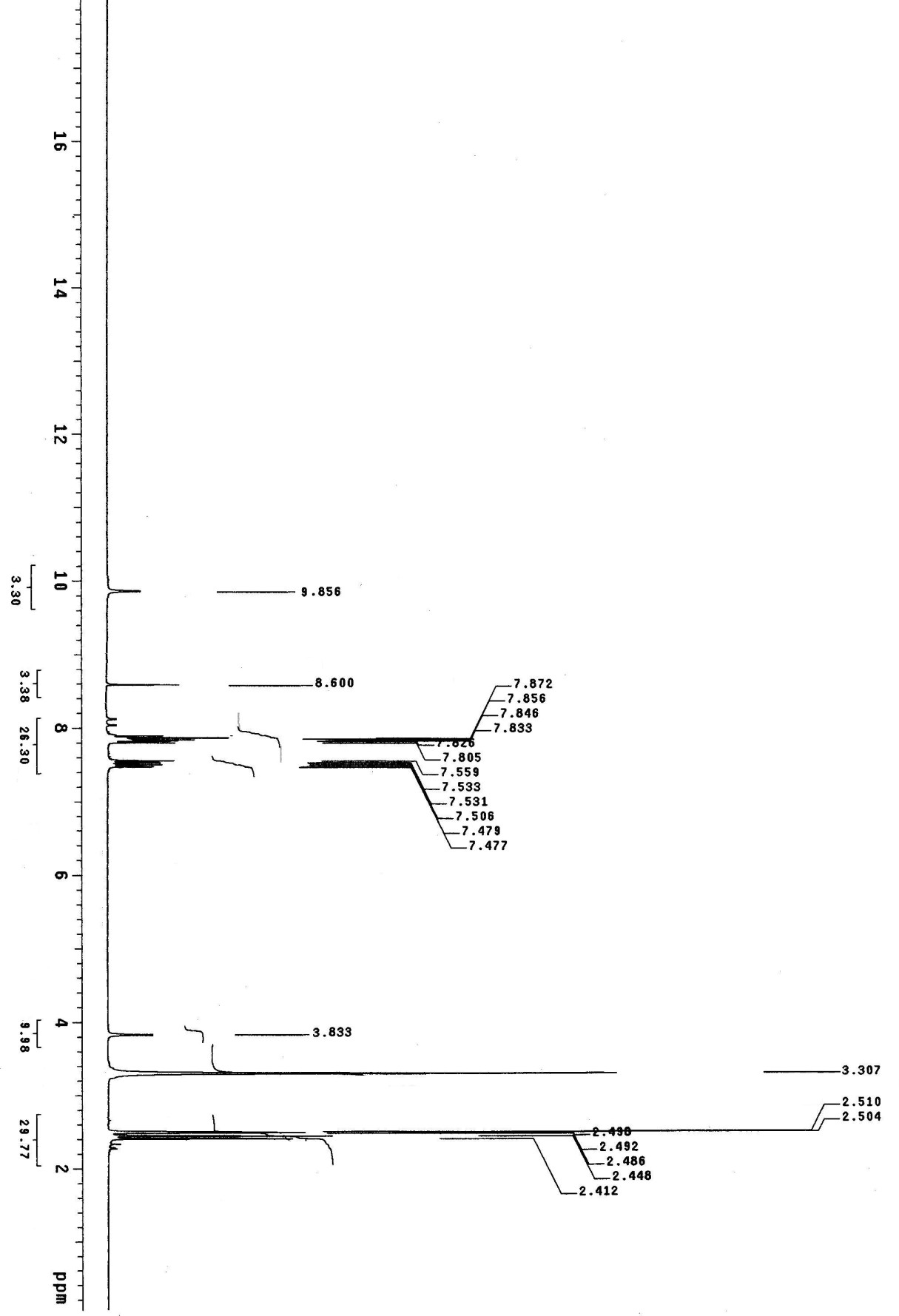


Compound 4 ^1^H-NMR


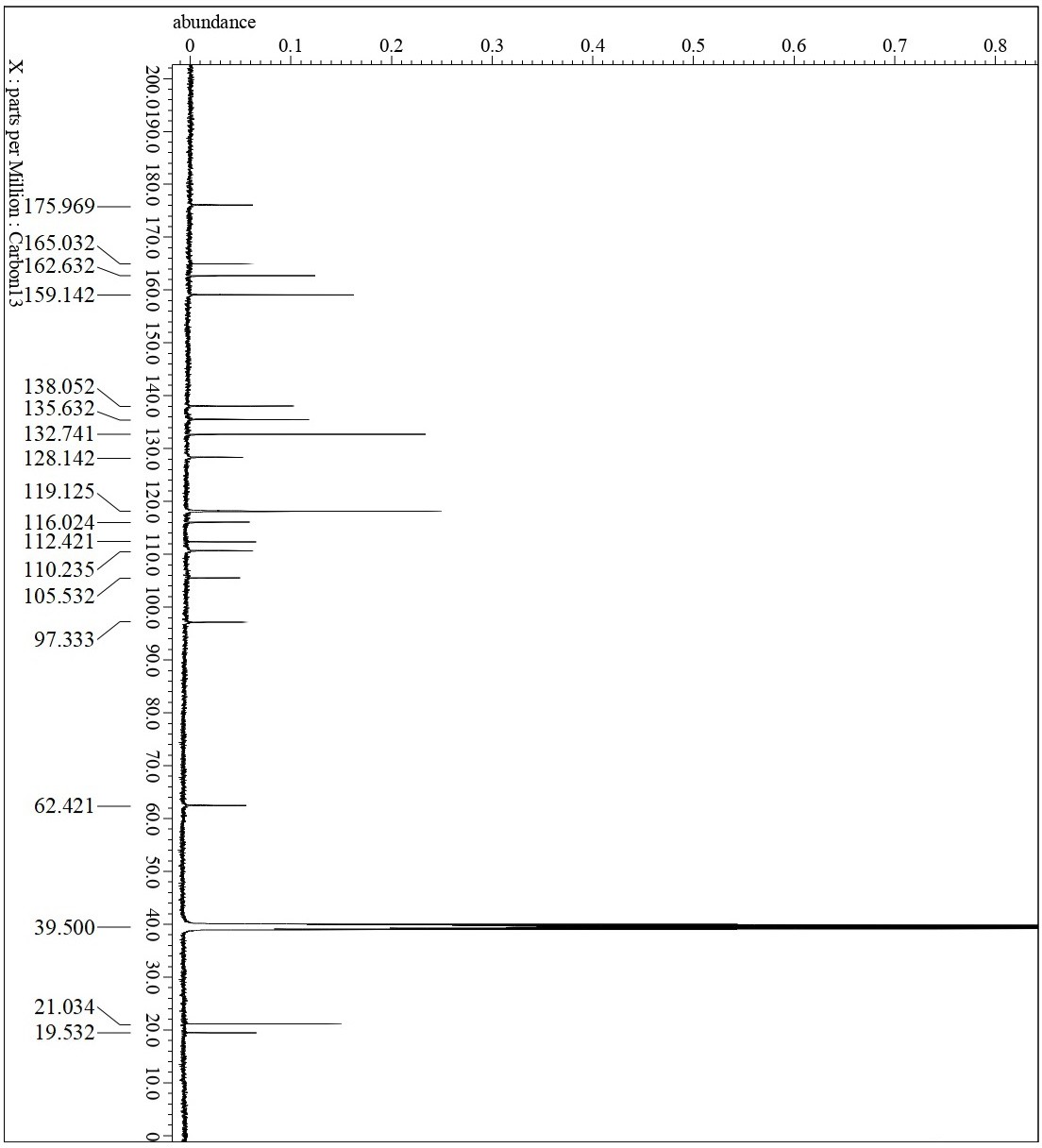


Compound 4 ^13^C-NMR


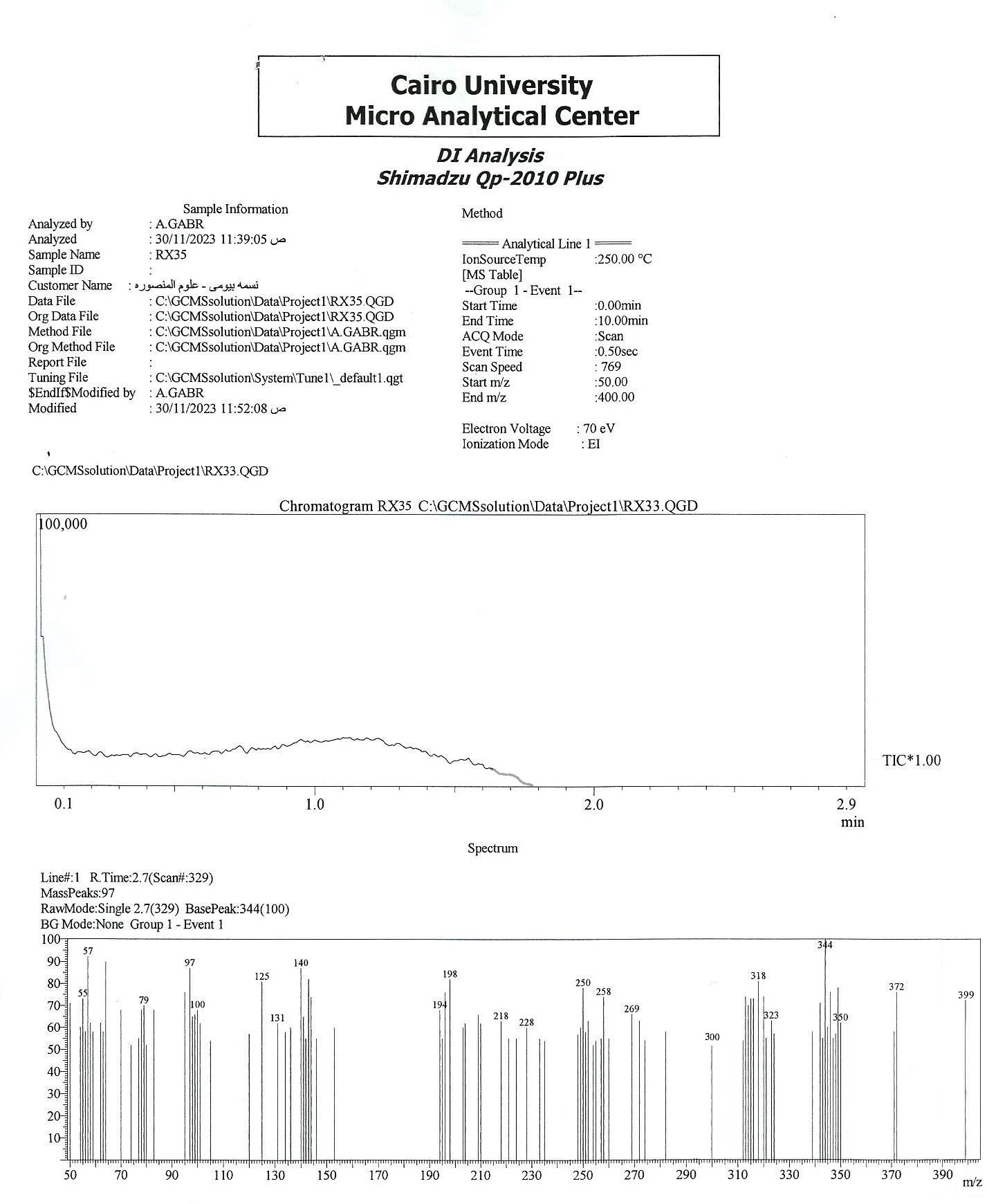


Compound 5 Mass spectrum


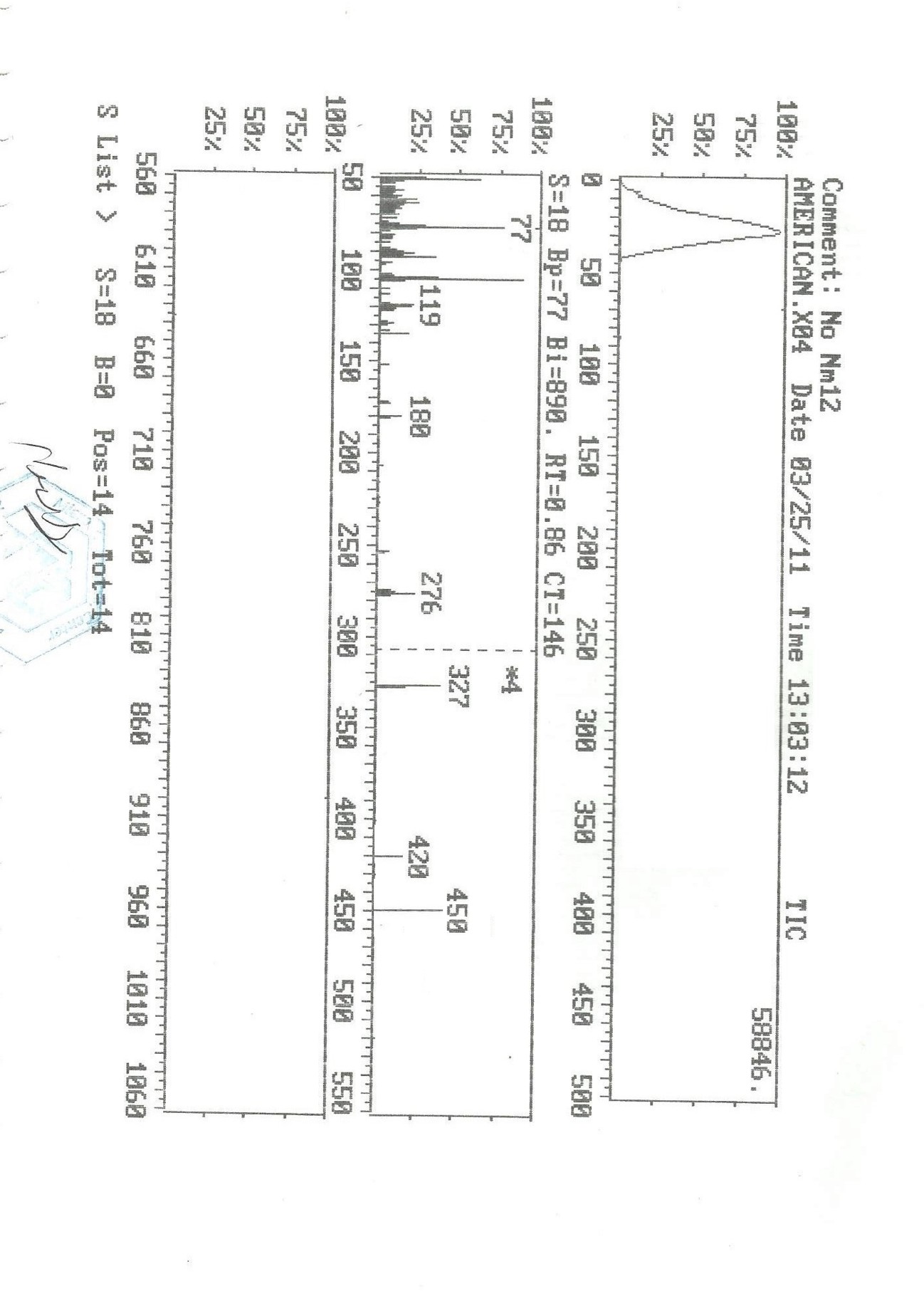


Compound 6 Mass spectrum


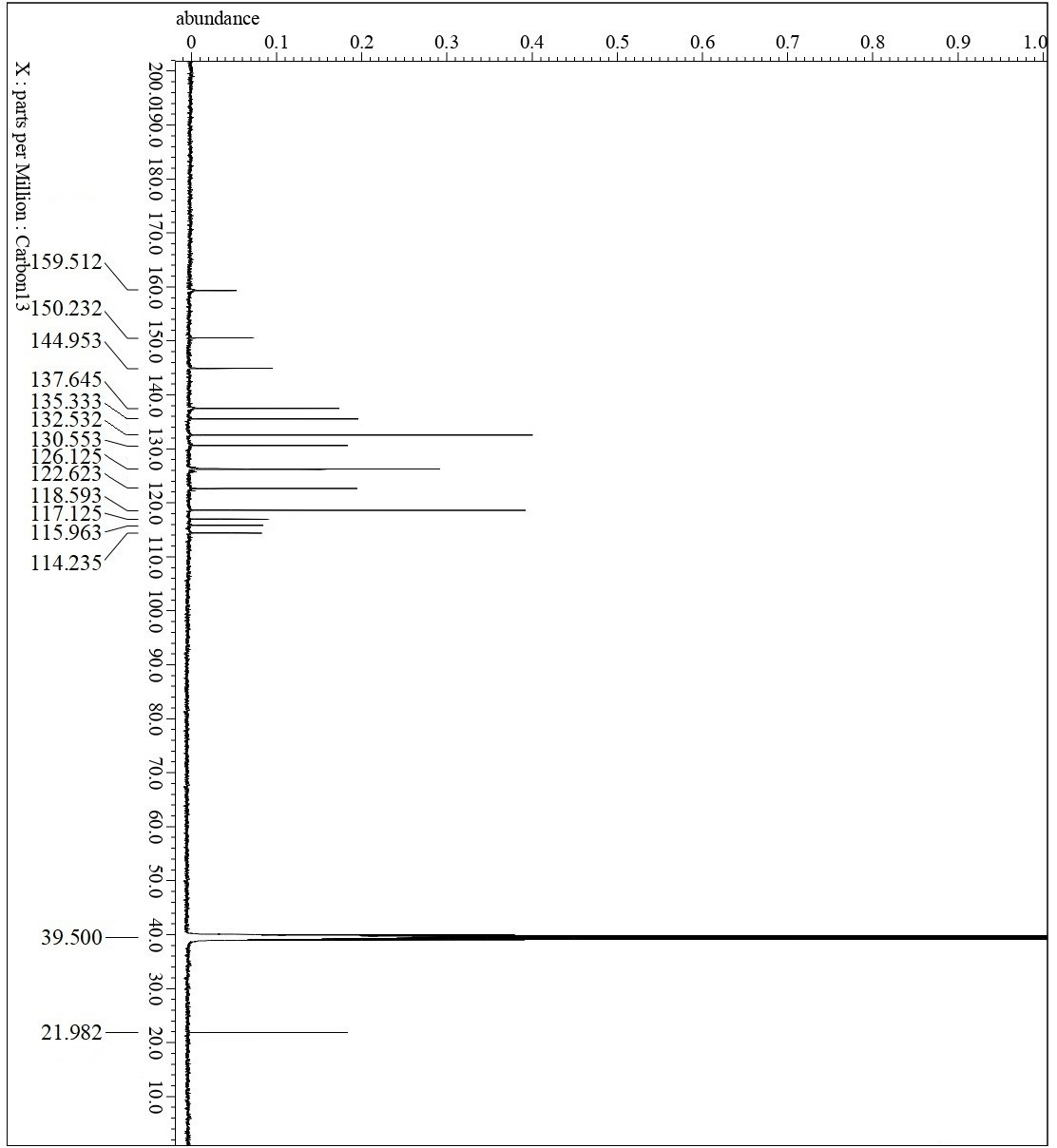


Compound 6 ^13^C-NMR


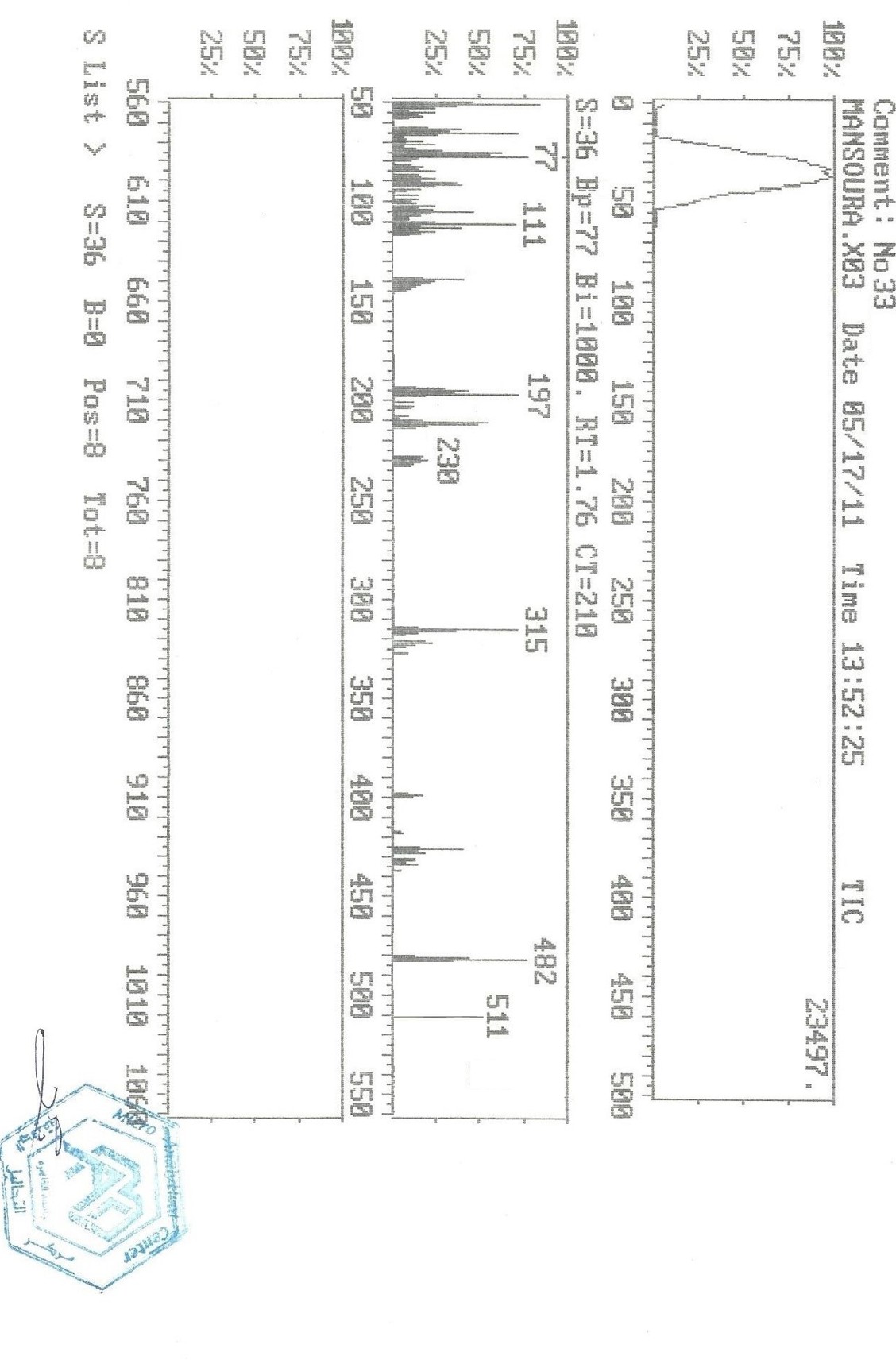


Compound 7 Mass spectrum


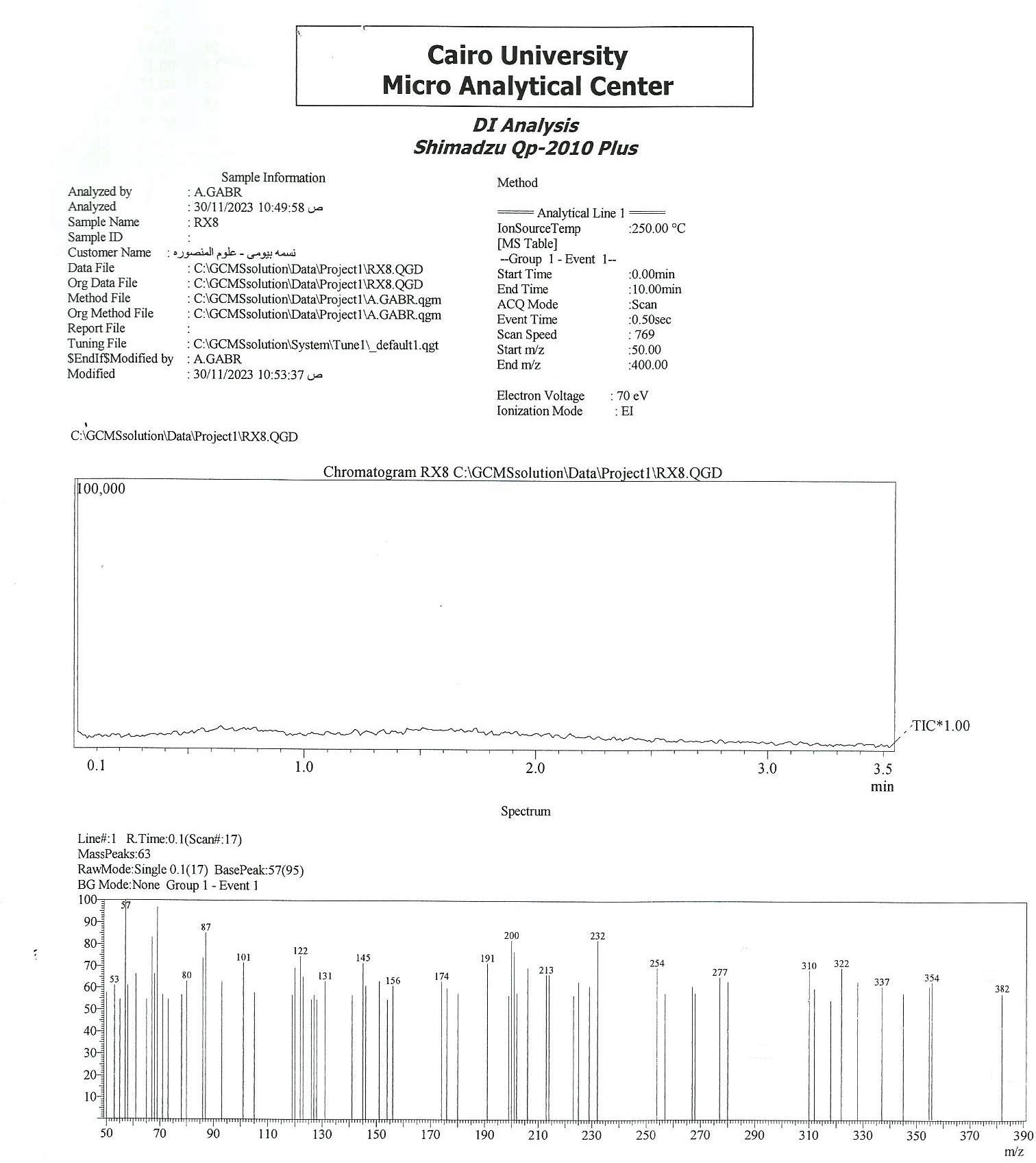


Compound 8a Mass spectrum


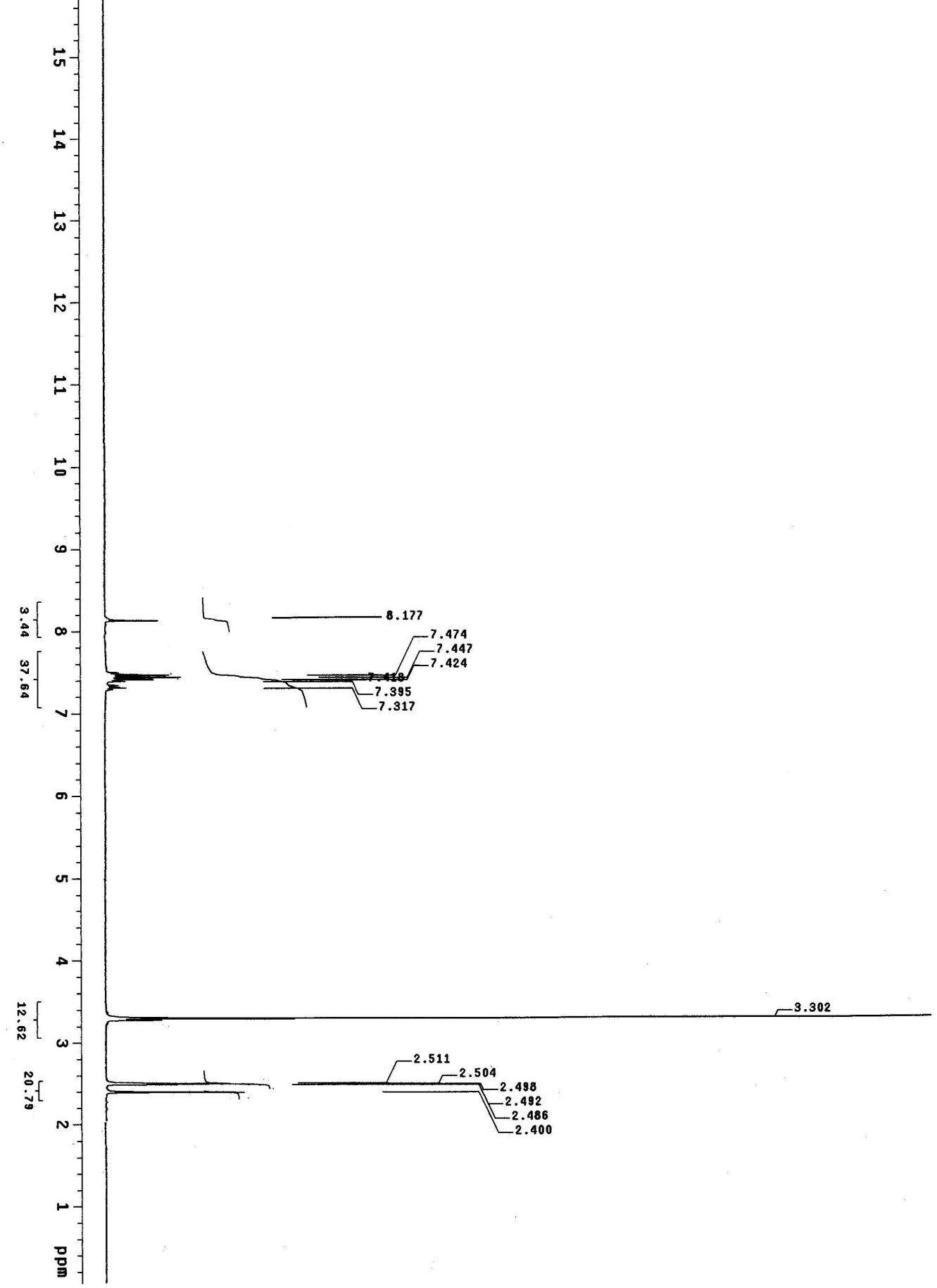


Compound 8a ^1^H-NMR


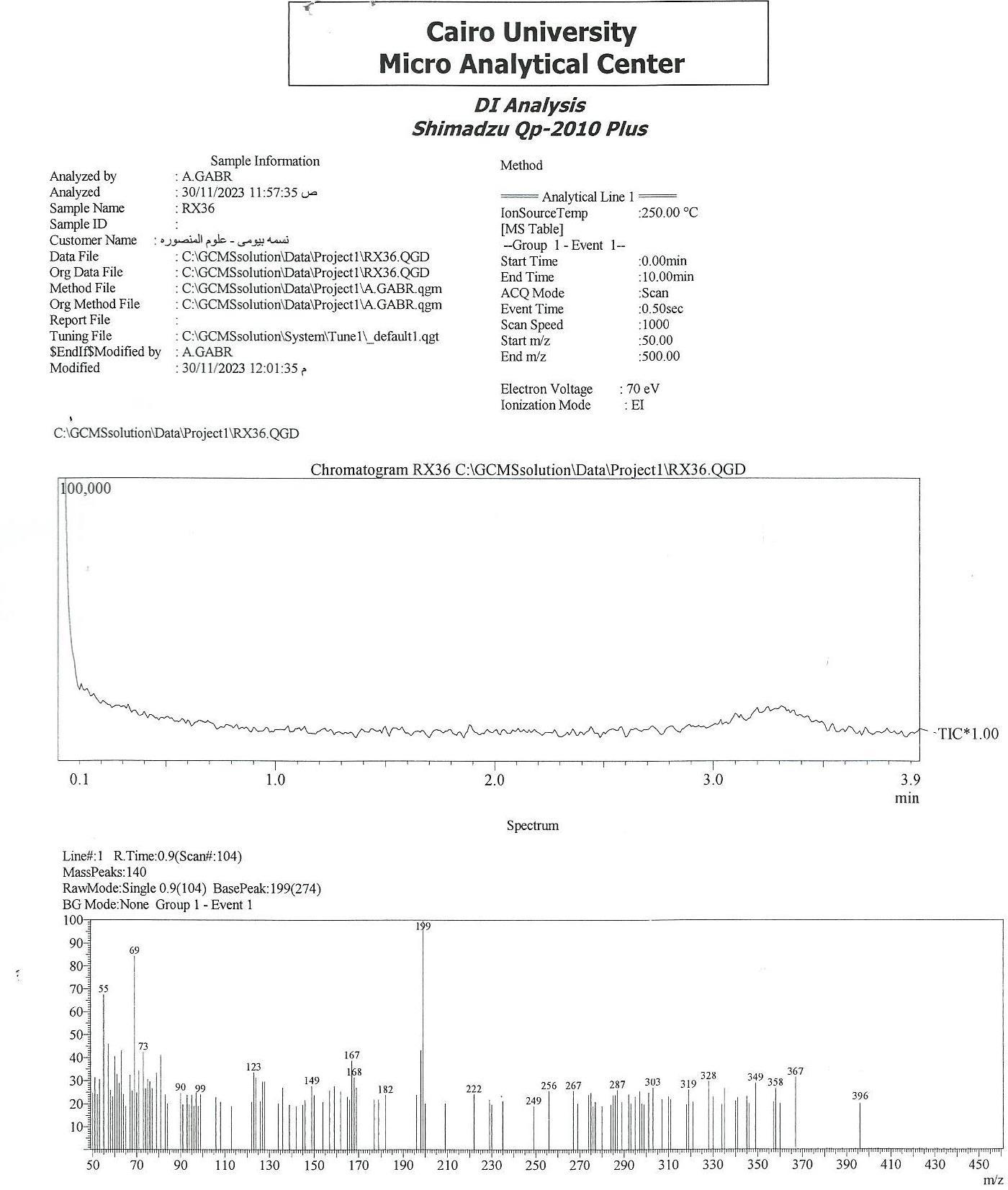


Compound 8b Mass spectrum


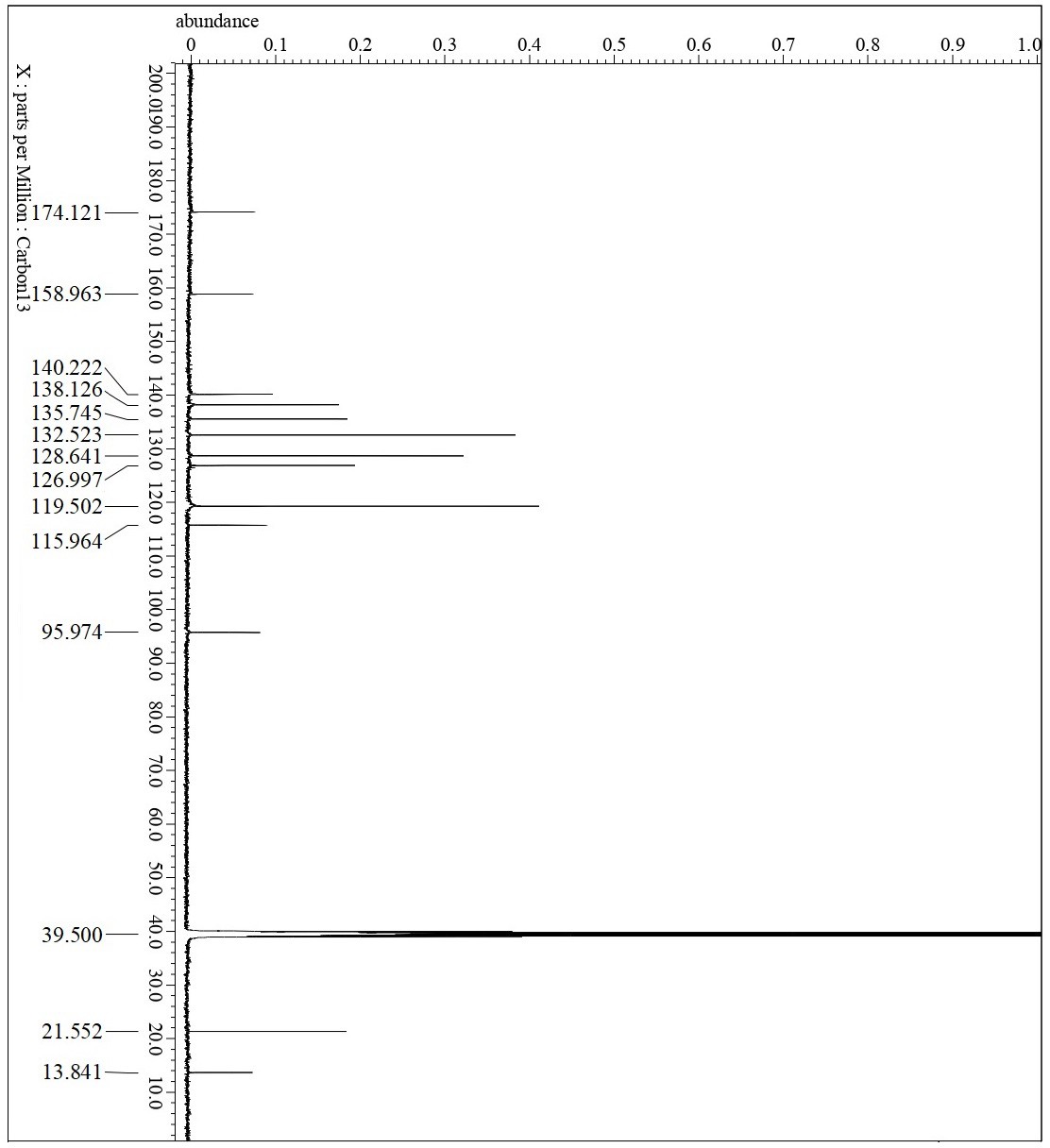


Compound 8b ^13^C-NMR


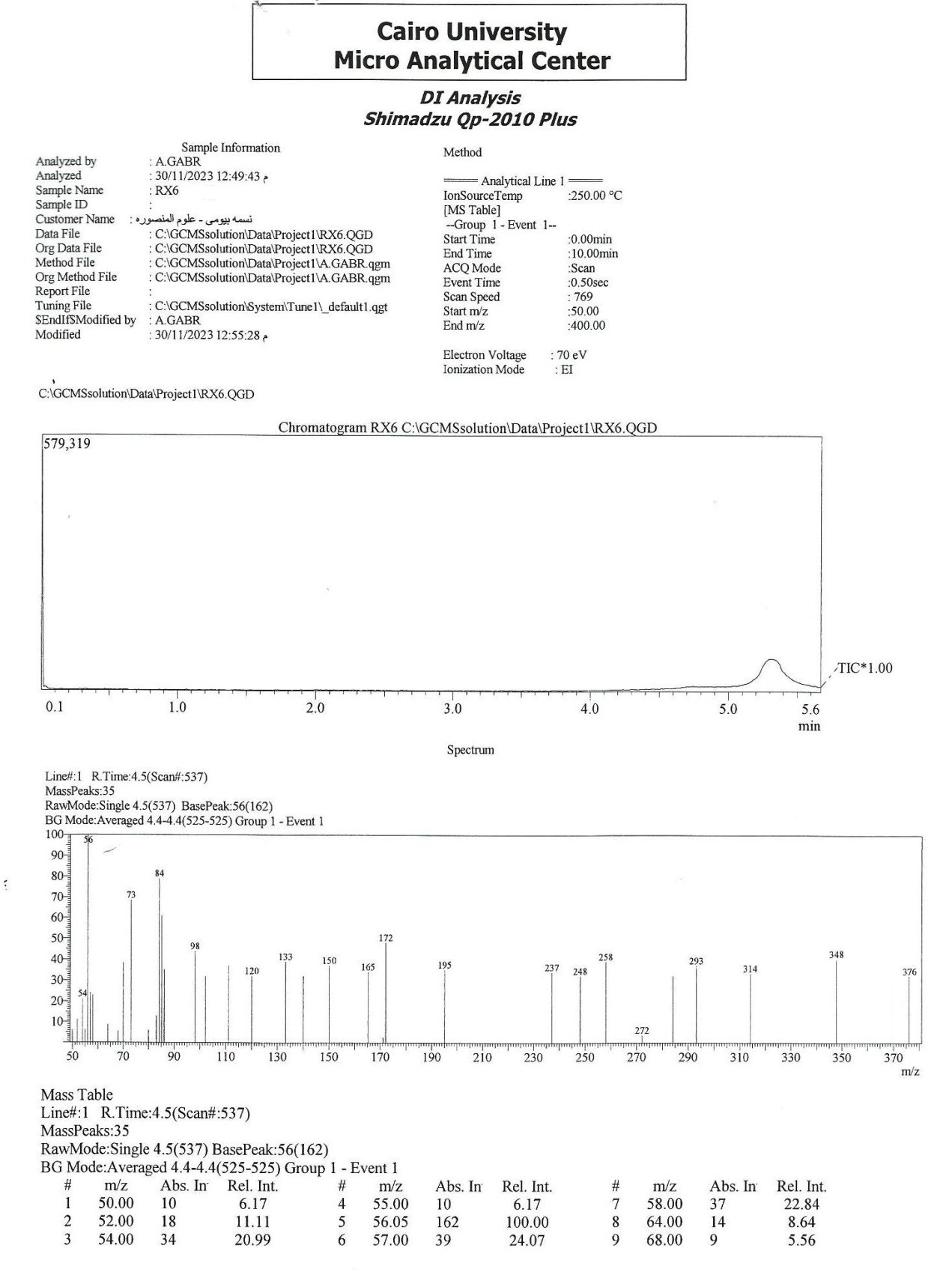


Compound 9 Mass spectrum


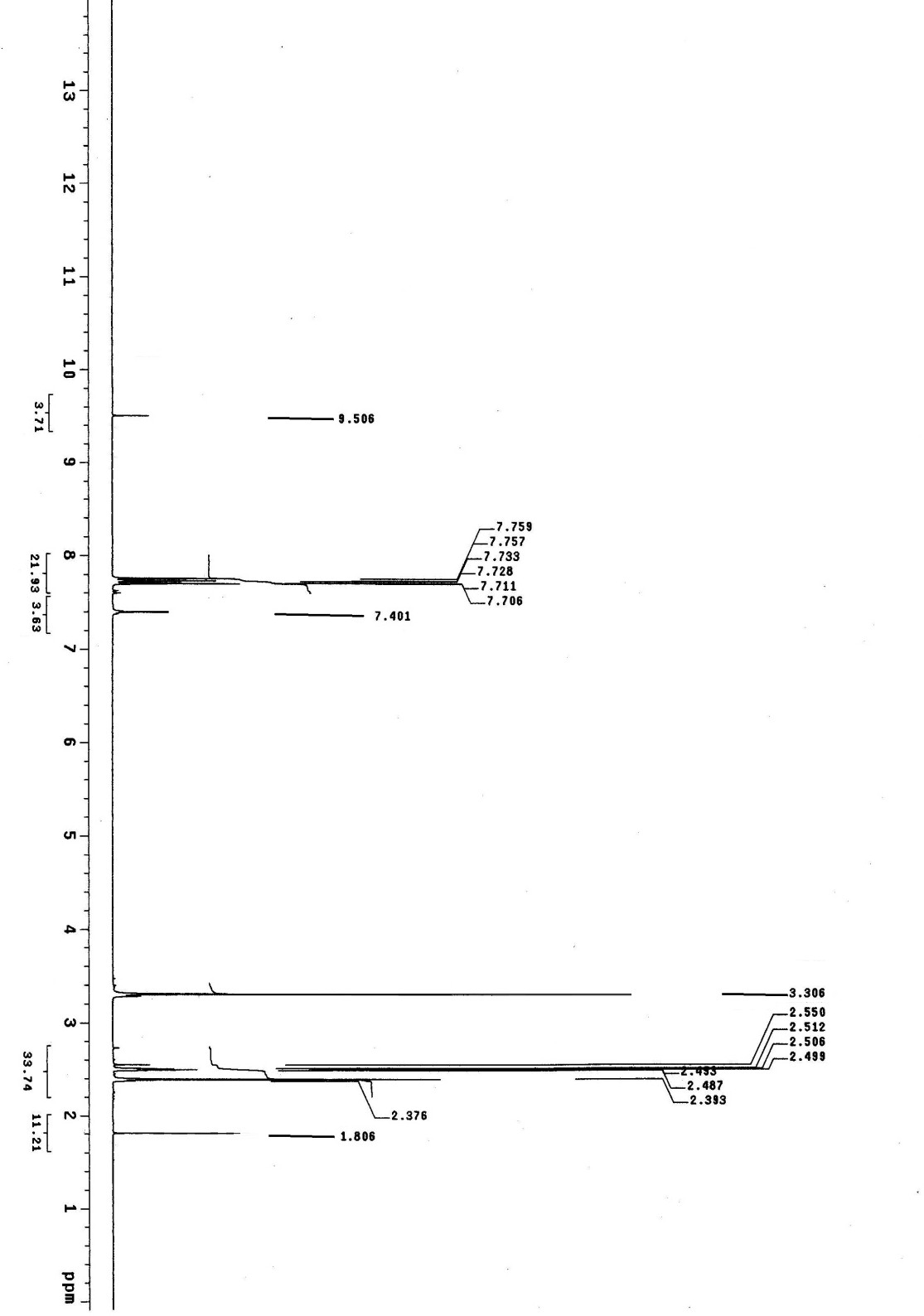


Compound 9 ^1^H-NMR


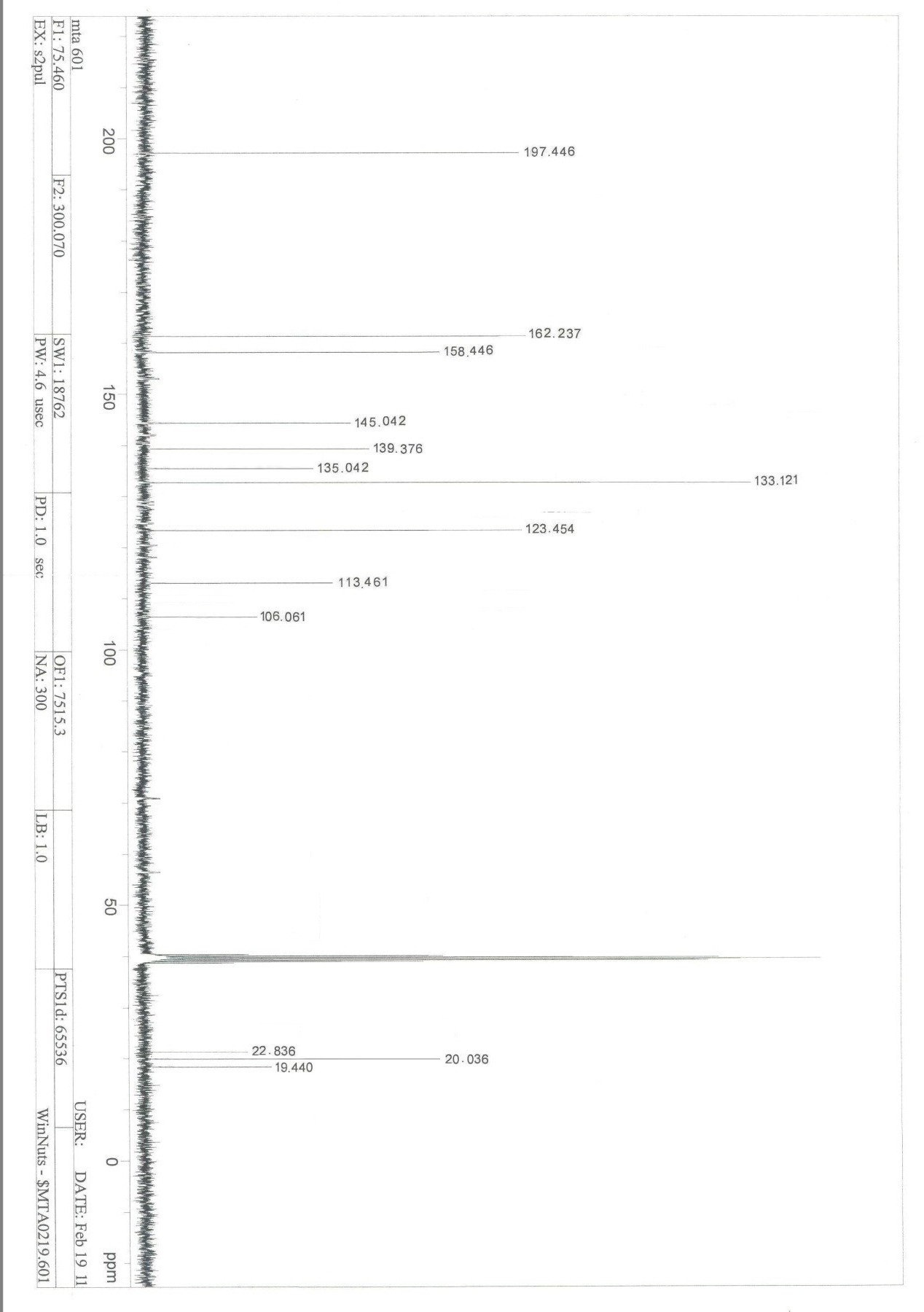


Compound 9 ^13^C-NMR


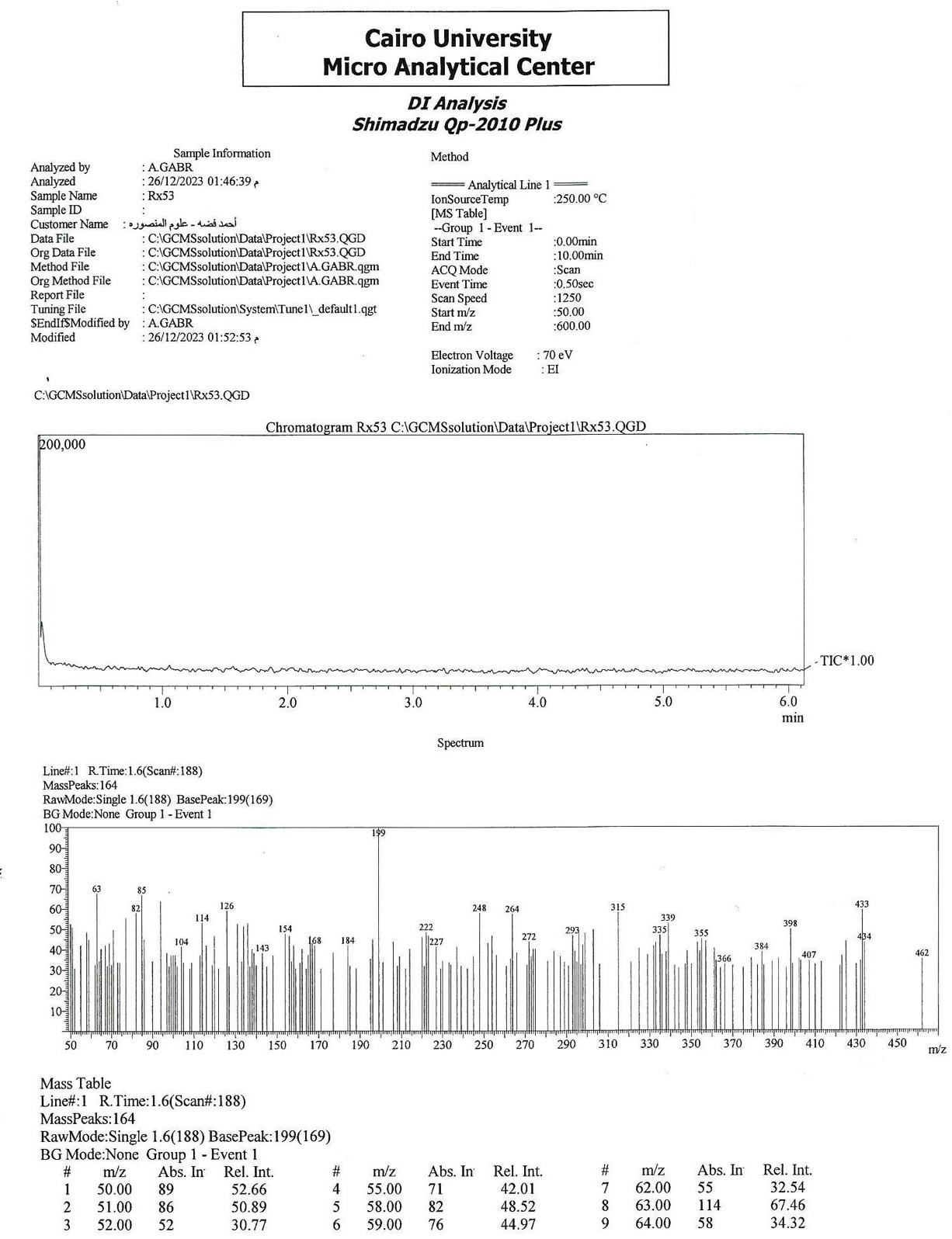


Compound 11 Mass spectrum


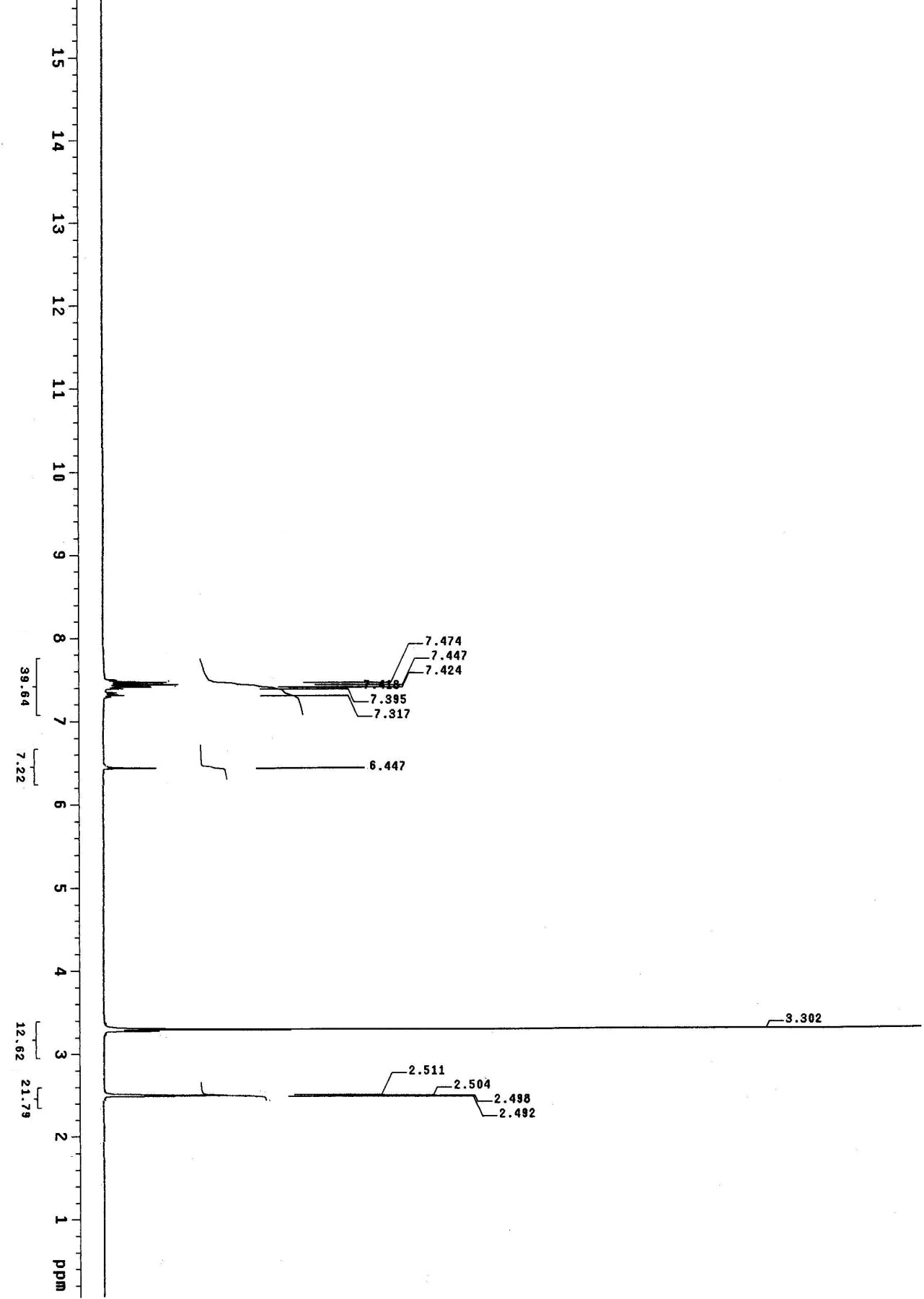


Compound 11 ^1^H-NMR


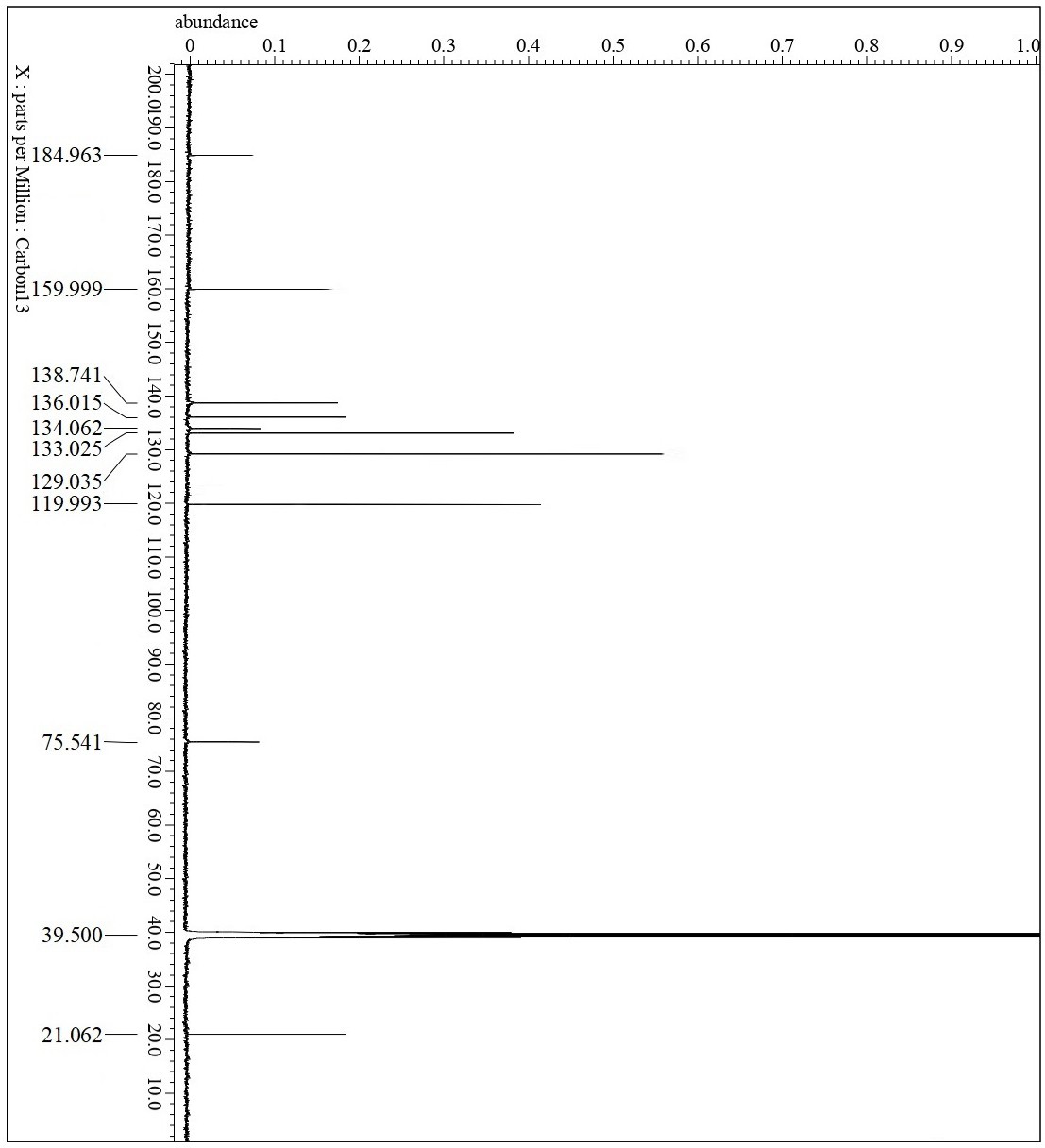


Compound 11 ^13^C-NMR


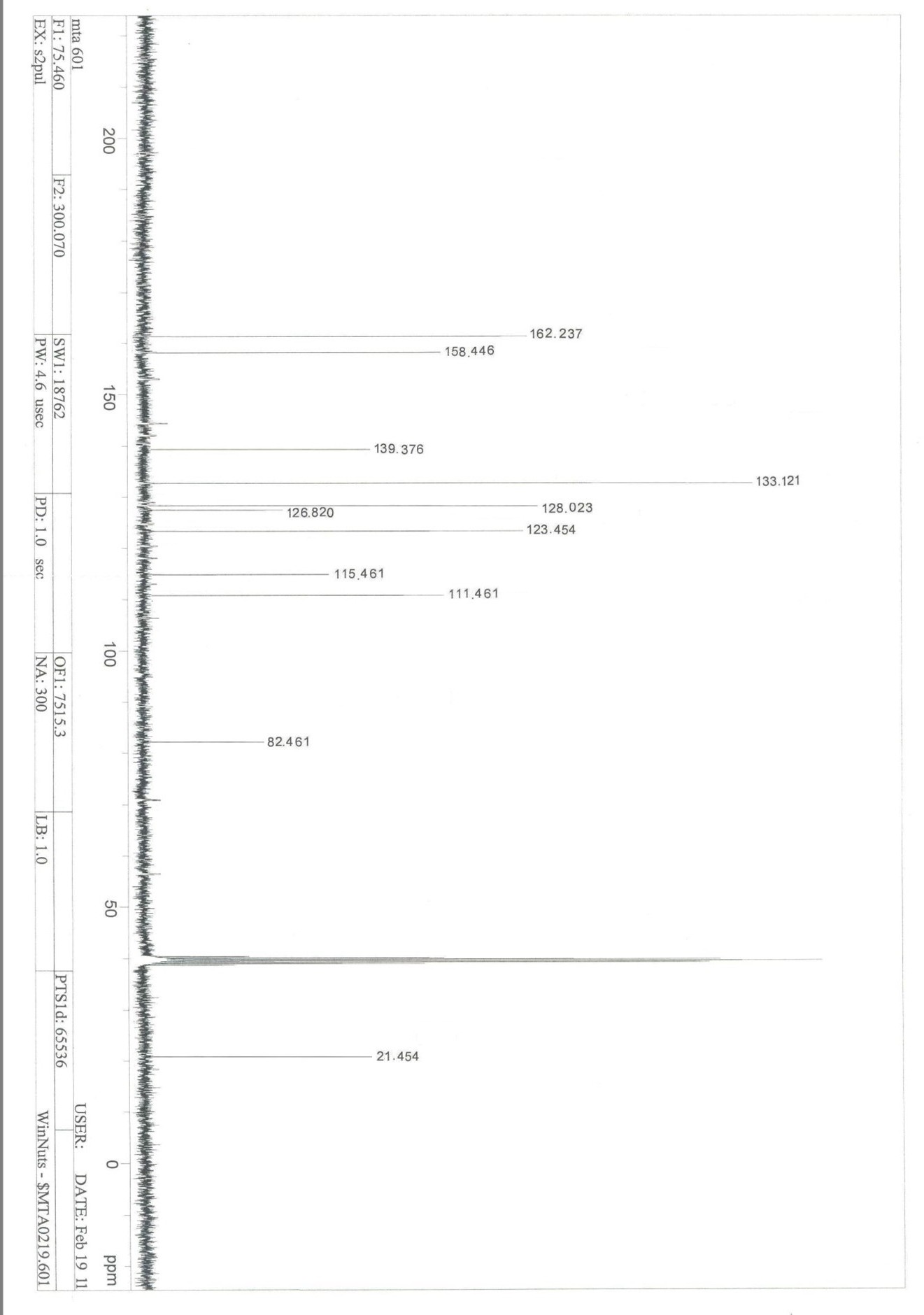


Compound 13 ^13^C-NMR


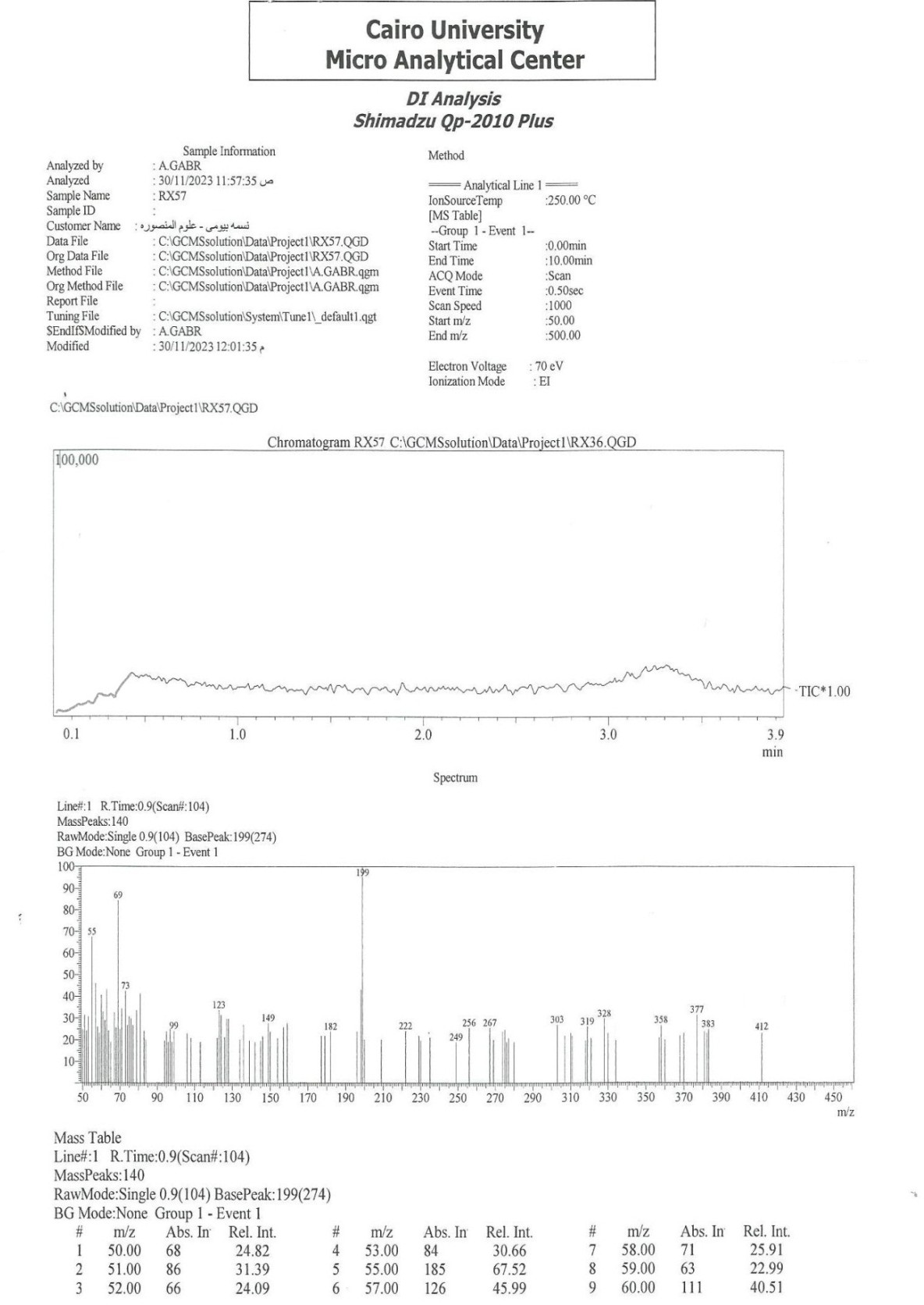


Compound 14 Mass spectrum


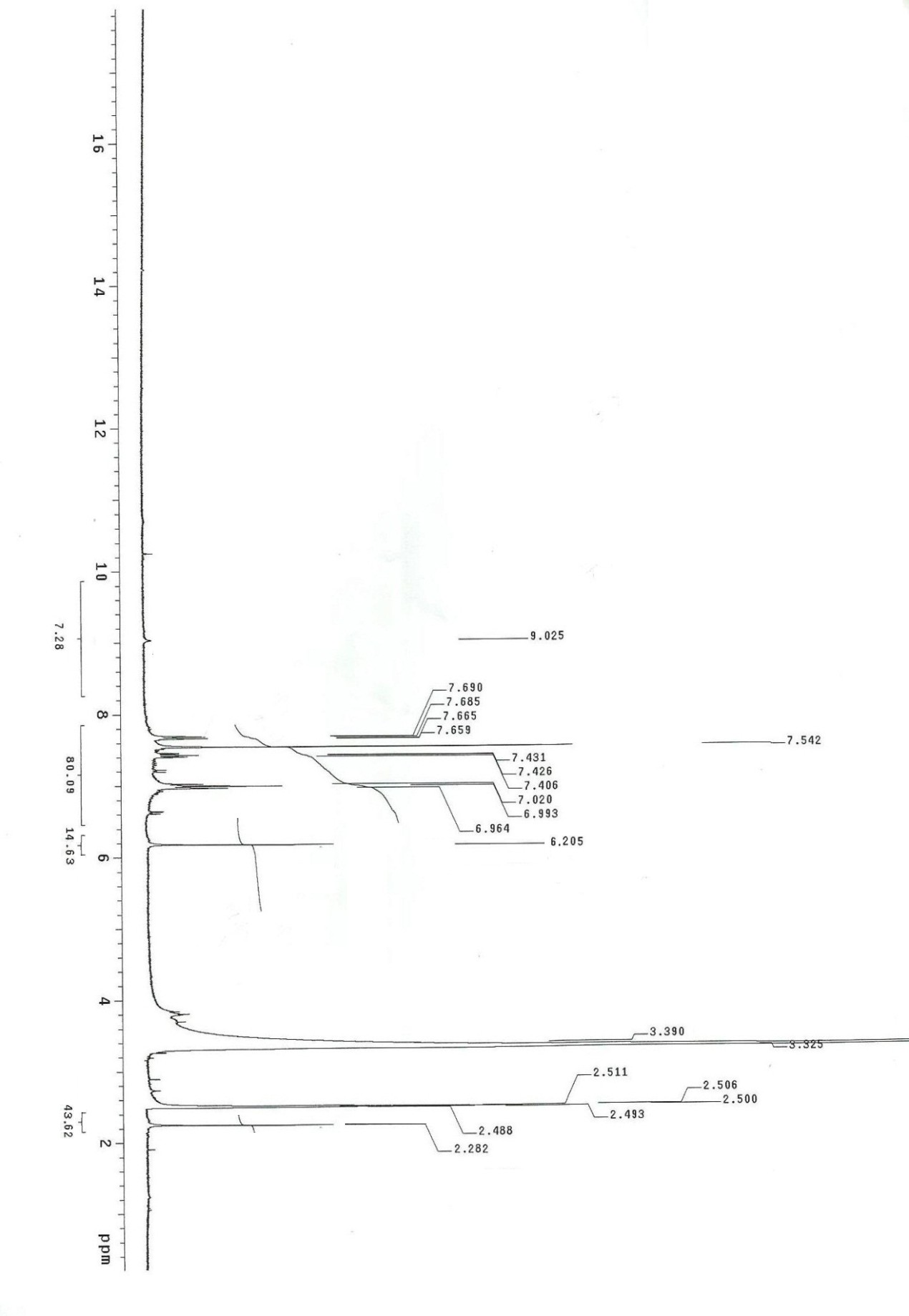


Compound 14 ^1^H-NMR


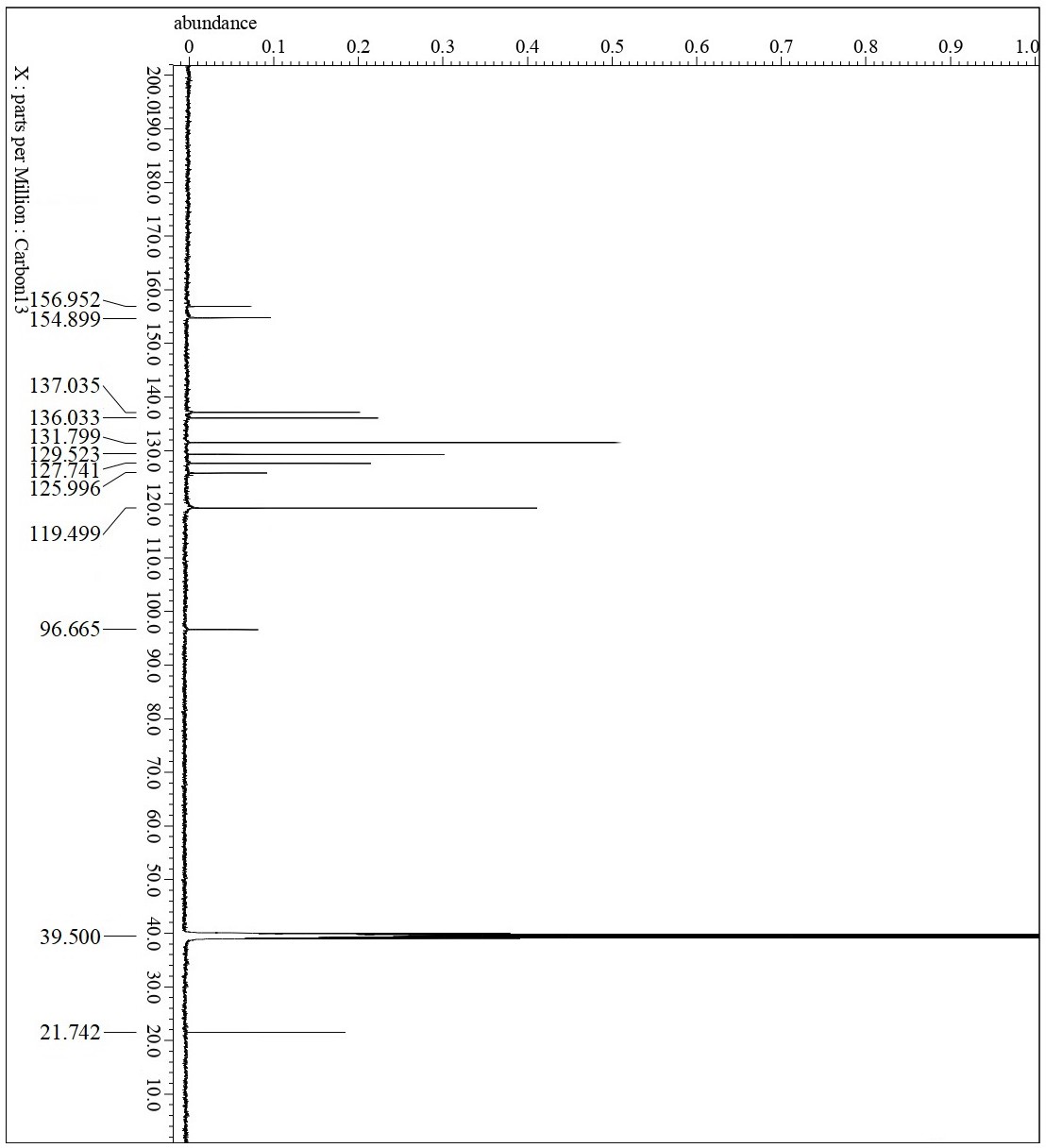


Compound 14 ^13^C-NMR
